# Multimodal analgesia in resource-limited settings: A comparative analysis of postoperative pain management strategies in Pakistan

**DOI:** 10.1371/journal.pgph.0005345

**Published:** 2025-12-19

**Authors:** Sana Wazir, Summaya Inayat, Muhammad Jawad Ullah, Gulmakay Zaman, Abdur Rehman, Faisal, Shallozan, Nuzhat Rahil

**Affiliations:** 1 Department of Oral and Maxillofacial Surgery, Medical Teaching Institute, Lady Reading Hospital, Peshawar, Pakistan; 2 Department of Chemistry, Shaheed Benazir University, Peshawar, Pakistan; 3 Department of Allied Health Sciences, Iqra National University, Peshawar, Pakistan; 4 North West School of Medicine, Peshawar, Pakistan; 5 Department of Gynecology, Hayatabad Medical Complex, Peshawar, Pakistan; 6 Kabul University of Medical Sciences, Kabul, Afghanistan; 7 Department of Ophthalmology Medical Teaching Institute, Lady Reading Hospital, Peshawar, Pakistan; University of Global Health Equity, RWANDA

## Abstract

Postoperative pain remains a critical yet often under-managed aspect of surgical recovery, influencing patient satisfaction, functional recovery, and quality of care. This study aimed to evaluate the comparative analysis of different postoperative pain management strategies and their impact on patient-reported outcomes in a tertiary care hospital in Pakistan. A cross-sectional analytical study was conducted among 431 surgical patients at a tertiary care facility. Data were collected on demographic variables, type of surgery, pain management strategy (monotherapy, opioid-based, multimodal, adjuvant), route of administration, analgesic frequency, patient satisfaction, side effects, and functional interference. Statistical analyses including chi-square tests, ANOVA, Pearson’s correlations, were performed. A p-value < 0.05 was considered statistically significant. Multimodal therapy was the most used strategy (29.9%), followed by opioid-based (27.6%) and monotherapy (26.7%). Patient satisfaction was significantly associated with pain management strategy (p < 0.001), with 36.9% reporting satisfaction and 20.2% very satisfied. While multimodal therapy showed lower mean pain scores than opioid-based regimens, the difference between these two groups was marginally non-significant (p = 0.060), suggesting a trend but not definitive superiority in terms of pain reduction. However, multimodal therapy was significantly associated with earlier mobilization compared to opioid-based regimens (p < 0.001). Significant correlations were observed between type of pain management and both pain score (r = -0.177, p = 0.000) and mobilization time (r = -0.116, p = 0.016). Preoperative anxiety and depression were present in 29.9% of participants and significantly associated with pain perception (p = 0.005). Multimodal pain management significantly improves postoperative outcomes by reducing pain intensity and enhancing functional recovery. Implementing standardized multimodal analgesia protocols may optimize care quality in surgical patients.

## Introduction

Postoperative pain remains a significant clinical challenge, with studies estimating that 20–40% of patients worldwide experience inadequate pain relief following surgery, leading to prolonged recovery, increased healthcare costs, and higher risks of chronic pain development [1–3]. While various pain management strategies exist, opioid-based regimens continue to dominate clinical practice despite their well-documented adverse effects, including nausea, respiratory depression, and potential for dependence [4,5]. In recent years, multimodal analgesia - combining opioids with non-opioid adjuvants like NSAIDs, gabapentinoids, and regional anesthesia techniques - has emerged as the gold standard in high-income countries, demonstrating superior pain control with reduced opioid requirements [6,7]. However, critical gaps remain in our understanding of how these approaches translate to low- and middle-income countries (LMICs), where resource constraints, cultural differences in pain perception, and variations in healthcare infrastructure may significantly impact outcomes [8–10].

The current literature exhibits several important limitations. First, most evidence supporting multimodal analgesia comes from Western populations, with minimal data from South Asian contexts where genetic, cultural, and pharmacological factors may alter treatment responses [11–13]. Second, few studies have examined the implementation challenges of multimodal approaches in resource-limited settings, where medication availability, cost considerations, and training limitations may affect feasibility. Third, while psychological factors like preoperative anxiety and depression are known to influence pain perception, their impact has been understudied in LMIC surgical populations. Finally, existing research predominantly focuses on short-term pain scores rather than functional recovery metrics or long-term outcomes like chronic pain prevention.

This study addresses these gaps by comprehensively evaluating postoperative pain management strategies in a Pakistani tertiary care center. We compare the effectiveness of four approaches - monotherapy, opioid-based regimens, multimodal analgesia, and adjuvant therapies - across multiple outcomes: pain intensity, functional recovery, patient satisfaction, and side-effect profiles. Our work represents the first systematic assessment of multimodal analgesia in this context while incorporating novel elements like gender-specific analyses and psychological factor assessments. By providing LMIC-specific evidence, we aim to inform the development of context-appropriate pain management protocols that balance efficacy, safety, and feasibility in resource-constrained environments. We hypothesize that multimodal analgesia will demonstrate superior outcomes compared to traditional opioid-centric approaches, with variations influenced by surgical type, psychological factors, and patient demographics.

Our findings have important implications for clinical practice and health policy in LMICs. As global healthcare systems strive to reduce opioid dependence while improving pain management, this study provides crucial data to guide protocol development in under-resourced settings. Furthermore, by identifying local barriers to effective pain control and highlighting population-specific response patterns, our work contributes to the growing movement toward personalized, context-sensitive postoperative care strategies. The integration of psychological assessments with clinical outcomes offers additional insights that could transform preoperative evaluation processes in similar healthcare environments worldwide. The primary research question guiding this study was: Among surgical patients in a resource-limited setting, how are postoperative pain management strategies associated with pain intensity and mobilization time? Secondary analyses examined patient satisfaction and adverse events.

## Methods

### Study design and setting

We conducted a cross-sectional comparative study at the surgical departments of different tertiary care hospitals [Lady Reading Hospital, Khyber Teaching Hospital and Hayatabad Medical Complex, Peshawar, Pakistan] in Pakistan from February 2023 to June, 2024. These hospitals serve a diverse urban population and performs a range of general and specialized surgeries.

### Ethics statement

The study protocol was reviewed and approved by the Research Ethical Committee of Iqra National University. Written informed consent was obtained from all participants prior to data collection. All procedures followed the principles outlined in the Declaration of Helsinki.

### Participants

The study included 431 adult patients (≥18 years) admitted for elective or emergency surgical procedures under general or spinal anesthesia. Eligible patients were enrolled consecutively using non-probability convenience sampling. Patients who were unconscious, critically ill, had psychiatric illness, or refused consent were excluded from the study.

### Data collection

The full survey instrument used for data collection, including demographic items, pain assessment scales, and satisfaction measures, is provided as S1 Table for transparency and to allow assessment of survey rigor and potential sources of bias. A pretested and validated questionnaire was used, consisting of demographic details, type of surgery performed, the analgesic regimen prescribed, pain intensity, and satisfaction level.

Pain intensity was measured using the Numerical Rating Scale (NRS), ranging from 0 (no pain) to 10 (worst possible pain). Pain assessment was conducted at three time points: within 6 hours postoperatively, on the first postoperative day, and on the third postoperative day. Patient satisfaction with pain management was recorded using a 5-point Likert scale ranging from “very dissatisfied” to “very satisfied.” Side effects were documented within 72 hours postoperatively from patient reports and clinical records, but causality to individual drug classes cannot be assumed given multimodal use in many cases. Hepatotoxicity events were recorded from clinician documentation in patient records, based on available biochemical or clinical evidence, but were not systematically measured in all patients.

#### Anxiety and depression status.

Preoperative anxiety and depression were assessed as part of standard clinical evaluations. However, **no specific validated anxiety/depression instrument** (e.g., HADS, PHQ-9, GAD-7) was used in this study. These factors were included as baseline covariates in adjusted models to account for potential confounding, but were not incorporated using formal instruments. Future studies may benefit from the use of validated tools to assess these psychological factors more rigorously.

#### Multiplicity control.

No formal multiplicity control, such as Bonferroni correction, was applied in this study. The primary reason for this decision was the focus on a single **primary endpoint** (postoperative pain NRS score at 24 hours), and the **secondary endpoints** (time-to-mobilization and opioid consumption) were considered **exploratory**. Given the relatively limited number of comparisons and the nature of the study as a preliminary exploration of different analgesic strategies, we did not implement multiplicity adjustments. The results from secondary outcomes are presented for descriptive purposes and should be interpreted as exploratory findings.

#### Pain scoring timing.

Pain scores were measured at multiple time points, including preoperative, 0–6 h, 6–24 h, and 24 hours post-surgery. The **primary pain outcome** was **pain NRS at 24 hours**, as it represents a clinically relevant measure of analgesic effectiveness in the immediate postoperative phase. While repeated pain scores were recorded at other time points, **24-hour pain NRS** was selected as the primary endpoint.

#### Repeated pain measurements.

Mixed-effects models for modeling pain trajectories were not implemented, as the study’s primary aim was to compare analgesic strategies at **24 hours postoperatively**, rather than assess pain over a prolonged period. Future research may explore pain trajectories more comprehensively.

#### Minimally important difference (MID).

Although **MID thresholds** are important for framing clinical significance, they were not incorporated in the current analysis. The focus of the study was on the comparison of analgesic strategies and their ability to reduce pain at 24 hours. Future studies should consider using **MID values** to further assess the clinical relevance of pain score differences.

### Pain management approaches

Patients were categorized based on the type of pain management received:

1. **Group A** – Monotherapy (e.g., paracetamol or NSAIDs alone)
2. **Group B** – Opioid-based regimens
3. **Group C** – Multimodal analgesia (combination of opioid and non-opioid drugs)
4. **Group D** – Adjuvant therapies (e.g., nerve blocks, regional anesthesia with pharmacologic support)

The type and frequency of analgesic administration, route (oral, intravenous, intramuscular), and any reported adverse effects were documented. Rescue analgesia, if administered, was recorded as a separate variable but did not alter the classification of the primary pain management group. For example, patients initially categorized under oral monotherapy but subsequently receiving intravenous opioid rescue remained in the monotherapy group, with rescue therapy documented separately. The primary outcomes were postoperative pain score (Numerical Rating Scale) and time to mobilization. Secondary outcomes included patient satisfaction and reported side effects.

#### Rescue analgesia.

Rescue therapy was captured as a separate variable and **did not** change the recorded *primary* exposure if given **after 24 hours**. If rescue occurred **within 24 hours** and altered class membership (e.g., oral non-opioid followed by IV opioid with overlap), the case was reclassified per the hierarchy above (typically into **True multimodal**). The frequency, route, and timing of rescue doses are summarized in S2 Table and descriptively in Table 3 (rescue use).

#### Agent-level exposure details.

For each participant, we abstracted **specific agents**, **dose (mg or mg/kg)**, **route** (oral/IV/IM/regional/neuraxial), **timing** (intraoperative; PACU/0–6 h; 6–24 h; > 24 h), and **dosing schedule** (bolus/PRN/infusion). Summary distributions (median [IQR] or % by category) are provided in S3 Table. Side-effects were captured within 72 hours as described previously; causality to an individual agent/class is **not** inferred in this observational design.

#### Exposure definition (mutually exclusive groups).

To ensure reproducible classification, analgesic exposures were defined a priori using drug class, route, and timing windows relative to surgery:

1. **Opioid-only**: systemic opioid(s) administered with **no non-opioid systemic analgesic** and **no regional/neuraxial** technique within the first **24 hours** post-surgery.
2. **Non-opioid-only**: systemic non-opioid analgesic(s) (e.g., acetaminophen/paracetamol, NSAIDs, gabapentinoids) with **no systemic opioid** and **no regional/neuraxial** within 24 hours.
3. **Regional + non-opioid adjuncts**: any **regional or neuraxial** technique (e.g., peripheral nerve block, local infiltration, epidural/spinal) with **non-opioid systemic** agents, and **no systemic opioid** within 24 hours.
4. **True multimodal**: **≥ 2 classes** administered with **temporal overlap** (any overlap within **0–6 hours**), **including** at least one systemic opioid **and** at least one non-opioid and/or regional/neuraxial modality within the first **24 hours** S2 Table.

### Pain management approaches (after exposure definitions)

#### Opioid normalization.

To enable interpretable comparisons, all opioid doses were converted to **morphine milligram equivalents (MME)** using route-appropriate perioperative equianalgesic factors S3 Table. We computed **total 0–24 h MME** as the primary exposure metric (intraoperative through 24 h) and **0–48 h MME** as a secondary metric when available. For multi-modal regimens, only opioid components contributed to MME; non-opioid agents were not converted.

#### Counting rules.

- **Bolus doses (IV/IM/PO):** sum of all administered doses × drug-specific factor.
- **PCA (patient-controlled analgesia):**
  - **Basal**: (basal rate, mg/h) × hours on PCA.
  - **Demand**: (dose per demand, mg) × **number of successful deliveries actually administered** (not attempts).
  - **Total PCA opioid** = basal + delivered on-demand.
- **Continuous infusion:** (infusion rate, mg/h) × hours infused.
- **Intraoperative fentanyl/remifentanil:** sum of all intraoperative opioid doses converted to MME and included in the 0–24 h window.
- **Neuraxial/systemic overlap:** neuraxial opioids (e.g., intrathecal/epidural morphine) were converted to MME with caution; **a sensitivity analysis** excluded neuraxial opioid from MME to assess robustness.
- **Rescue therapy:** rescue opioid doses within 0–24 h were included in total MME; this did not alter the patient’s mutually exclusive exposure group classification (defined a priori), which is based on class overlap rules.
- **Units and rounding:** microgram doses were converted to mg prior to applying factors; totals were reported to 1 decimal place.

#### Primary variables derived.

- **Total MME 0–24 h (mg)** and **0–48 h (mg)** (if available).
- **PCA present (yes/no)**; **PCA total MME**; **infusion total MME** (mg).

#### Statistical use.

We summarized MME medians (IQR) by exposure group and incorporated **MME as a covariate** in adjusted models:

- Pain NRS (continuous): linear model/ANCOVA = *NRS* ~ exposure group + **MME** + surgery type + age + sex + preop anxiety.
- Time to mobilization (hours): linear model with the same covariates.
- Sensitivity: models excluding neuraxial opioid from MME and models restricted to non-neuraxial systemic opioid only.

### Statistical analysis

Data were analyzed using SPSS version 25.0 (IBM Corp., Armonk, NY). Continuous variables were summarized using means and standard deviations, while categorical variables were expressed as frequencies and percentages. Comparisons between groups were made using one-way ANOVA for continuous variables and chi-square tests for categorical variables. A p-value of <0.05 was considered statistically significant.

## Results

Table 1 summarizes the descriptive statistics for three key continuous variables among the study participants (N = 431). The mean postoperative pain score measured by the Numerical Rating Scale (NRS) was 4.21 (SD = 1.53), with scores ranging from 0 to 10, indicating a moderate level of pain reported after surgery. The time to mobilization post-surgery varied widely, with a mean duration of 37.77 hours (SD = 12.08), ranging from 10.10 to 65.90 hours. The variable “previous history of chronic pain,” coded as 1 (No) and 2 (Yes), had a mean value of 1.72 (SD = 0.44), indicating that most participants (approximately 72%) reported a prior history of chronic pain. These continuous measures provide a foundational understanding of postoperative recovery patterns and are used in further inferential analysis to assess the effectiveness of different pain management strategies. We normalized all opioid exposures to **0–24 h MME** (median [IQR]) and, where available, **0–48 h MME** S3 Table. As expected, MME was highest in the **opioid-only** group, intermediate in **true multimodal**, and near-zero in **non-opioid-only** and **regional + non-opioid** groups.

**Table 1:** Descriptive statistics of postoperative pain, mobilization time, and chronic pain history (N = 431).

	N	Minimum	Maximum	Mean	Std. Deviation
Postoperative Pain Score NRS	431	.00	10.00	4.21	1.53
Type To Mobilization Postsurgery	431	10.10	65.90	37.77	12.08

### Sociodemographic characteristics of participants

Table 2 presents the sociodemographic distribution of the study population (N = 431). Among the participants, females constituted a slight majority, representing 49.9% (n = 215), followed by males at 45.5% (n = 196), while 4.6% (n = 20) of respondents chose not to disclose their gender.

**Table 2:** Distribution of participants by gender and age group (N = 431).

Variables	Details	Frequency	Percentage
Gender	Male	196	45.5
	Female	215	49.9
	Prefer not to mention	20	4.6
Age Group	19-28 Years	97	22.5
	29-38 Years	120	27.8
	39-48 Years	107	24.8
	49-58 Years	58	13.5
	More than 59 Years	49	11.4

The age distribution showed that the largest group was between 29–38 years (27.8%, n = 120), followed by those aged 39–48 years (24.8%, n = 107), and 19–28 years (22.5%, n = 97). Participants aged 49–58 years and above 59 years accounted for 13.5% (n = 58) and 11.4% (n = 49), respectively. These findings highlight a relatively young to middle-aged adult population undergoing surgery and participating in the study.

### Clinical characteristics, pain management strategies, and patient-reported outcomes

Table 3 provides detailed clinical characteristics and treatment-related variables for the surgical cohort (N = 431). The most common surgical procedure was maxillofacial trauma reconstruction, reported in 14.6% (n = 63) of cases, followed by post-DCR (dacryocystorhinostomy) in 13.2% (n = 57), corneal laceration repair (12.5%, n = 54), and lid laceration repair (10.7%, n = 46). Other surgeries included post-exenteration (10.9%, n = 47), post-evisceration (9.0%, n = 39), repair of globe rupture and ORIF of jaw fractures (both 9.7%, n = 42), and miscellaneous procedures (9.5%, n = 41).

**Table 3:** Distribution of clinical, pain management, and patient outcome variables (N = 431).

Variables	Details	Frequency	Percentage
Type of Surgery	Post DCR (Dacryocystorhinostomy)	57	13.2
	Post Evisceration	39	9.0
	Temporomandibular joint surgery and Impacted tooth Surgery	47	10.9
	Post Repair of Corneal Laceration	54	12.5
	Repair of Globe Rupture	42	9.7
	Lid Laceration Repair	46	10.7
	ORIF (Open Reduction and Internal Fixation) of Jaw Fractures	42	9.7
	Maxillofacial Trauma Reconstruction	63	14.6
	Others	41	9.5
Type of pain management	Monotherapy	115	26.7
	Opioid-based	119	27.6
	Multimodal	129	29.9
	Adjuvant therapy	68	15.8
Route of administration	Oral	141	32.7
	IV	167	38.7
	IM	32	7.4
	Regional	91	21.1
Frequency of analgesic used	Once daily	64	14.8
	Twice daily	150	34.8
	PRN (as needed)	33	7.7
	Continuous infusion	184	42.7
Patient satisfaction	Very Dissatisfied	9	2.1
	Dissatisfied	43	10.0
	Neutral	133	30.9
	Satisfied	159	36.9
	Very Satisfied	87	20.2
Side effect	Nausea/Vomiting	81	18.8
	Constipation	61	14.2
	Sedation/Dizziness	32	7.4
	GI Upset/Bleeding	67	15.5
	Respiratory Issues	13	3.0
	Hepatotoxicity	17	3.9
	None	160	37.1
Rescue Analgesia Used	Yes	123	28.5
	No	308	71.5
Pain interference in function	No interference	104	24.1
	Mild interference	141	32.7
	Moderate interference	139	32.3
	Severe interference	38	8.8
	Complete inability	9	2.1
Preop anxiety and depression	Yes	129	29.9
	No	302	70.1

In terms of pain management, multimodal therapy was the most frequently used strategy (29.9%, n = 129), followed closely by opioid-based regimens (27.6%, n = 119), and monotherapy (26.7%, n = 115), while adjuvant therapies such as nerve blocks were administered to 15.8% (n = 68). The route of analgesic administration was predominantly intravenous (38.7%, n = 167), followed by oral (32.7%, n = 141), regional (21.1%, n = 91), and intramuscular (7.4%, n = 32). The most frequent dosing regimen was continuous infusion (42.7%, n = 184), followed by twice-daily administration (34.8%, n = 150), once daily (14.8%, n = 64), and PRN (as needed) use (7.7%, n = 33).

Regarding patient satisfaction, 36.9% (n = 159) were satisfied and 20.2% (n = 87) were very satisfied with their pain management, while 30.9% (n = 133) remained neutral. Only 2.1% (n = 9) were very dissatisfied. Among reported side effects, nausea/vomiting (18.8%, n = 81), gastrointestinal upset/bleeding (15.5%, n = 67), and constipation (14.2%, n = 61) were the most common. Notably, 37.1% (n = 160) reported no adverse effects.

Rescue analgesia was required in 28.5% (n = 123) of cases, while 71.5% (n = 308) managed without additional pain relief. In terms of functional interference due to pain, mild (32.7%, n = 141) and moderate (32.3%, n = 139) interference were most commonly reported, with 24.1% (n = 104) reporting no interference, and only 2.1% (n = 9) experiencing complete inability to function. Finally, preoperative anxiety or depression was reported in 29.9% (n = 129) of participants.

**Agent-level details, dose distributions, routes, and timing windows** for each exposure group (including rescue therapy) are summarized in S2 Table, facilitating appraisal of clinical heterogeneity across groups.

### Gender-wise comparison of surgical, analgesic, and outcome variables

Table 4 outlines the gender-wise distribution of key clinical, therapeutic, and outcome variables among the study participants (N = 431) and presents the results of statistical comparisons using chi-square tests. Among the surgical categories, no statistically significant association was found between gender and the type of surgery performed (p = 0.224). Similarly, no significant gender-based differences were observed in the type of pain management received (p = 0.495) or the route of analgesic administration (p = 0.329).

**Table 4:** Gender-wise distribution and association of surgical characteristics, analgesic use, and patient.

Variable	Details	Gender			P value
		Male	Female	Not prefer to mention	
Type of surgery	Post DCR (Dacryocystorhinostomy)	22	33	2	0.224
	Post Evisceration	26	11	2	
	Temporomandibular joint surgery and Impacted tooth Surgery	23	23	1	
	Post Repair of Corneal Laceration	23	29	2	
	Repair of Globe Rupture	17	21	4	
	Lid Laceration Repair	23	22	1	
	ORIF (Open Reduction and Internal Fixation) of Jaw Fractures	24	17	1	
	Maxillofacial Trauma Reconstruction	22	37	4	
	Others	16	22	3	
Type Of Pain Management	Monotherapy	53	57	5	0.495
	Opioid-based	52	63	4	
	Multimodal	57	62	10	
	Adjuvant therapy	34	33	1	
Route Of Administration	Oral	68	65	8	0.329
	IV	77	84	6	
	IM	8	22	2	
	Regional	43	44	4	
Frequency Of Analgesic Used	Once daily	23	34	7	0.169
	Twice daily	68	76	6	
	PRN (as needed)	18	14	1	
	Continuous infusion	87	91	6	
Patient Satisfaction	Very Dissatisfied	4	5	0	0.680
	Dissatisfied	24	17	2	
	Neutral	61	68	4	
	Satisfied	67	81	11	
	Very Satisfied	40	44	3	
Side Effect	Nausea/Vomiting	42	35	4	0.796
	Constipation	26	32	3	
	Sedation/Dizziness	13	19	0	
	GI Upset/Bleeding	30	33	4	
	Respiratory Issues	6	7	0	
	Hepatotoxicity	9	6	2	
	None	70	83	7	
Rescue_Analgesia_Used	Yes	56	66	1	0.05
	No	140	149	19	
Pain Interference In Function	No interference	42	57	5	0.364
	Mild interference	60	73	8	
	Moderate interference	74	58	7	
	Severe interference	17	21	0	
	Complete inability	3	6	0	
Preop Anxiety Depression	Yes	72	49	8	0.005
	No	124	166	12	
Previous History Of Chronic Pain	Yes	55	56	7	0.663
	No	141	159	13	

The frequency of analgesic dosing also did not differ significantly across genders (p = 0.169), with continuous infusion being the most common mode across all groups. Patient satisfaction levels were comparable between males and females (p = 0.680), with the majority reporting either satisfaction or neutrality.

Side effect profiles, including nausea/vomiting, constipation, sedation, and GI upset, also showed no statistically significant gender difference (p = 0.796). However, rescue analgesia use was marginally associated with gender, showing borderline significance (p = 0.050), with females slightly more likely to require additional pain relief (66 vs. 56 cases).

Pain interference in functional capacity was not significantly different across genders (p = 0.364), although moderate and mild levels were commonly reported in both groups. A statistically significant association was observed between preoperative anxiety/depression and gender (p = 0.005), with a higher number of males (n = 72) reporting symptoms compared to females (n = 49). No significant gender differences were noted in the previous history of chronic pain (p = 0.663).

### Association of pain management strategies with demographic, clinical, and outcome variables

Table 5 presents the cross-tabulation of demographic and clinical variables with the type of postoperative pain management strategy received, categorized as monotherapy, opioid-based, multimodal, and adjuvant therapy. No statistically significant associations were observed between the type of pain management and gender (p = 0.495) or age group (p = 0.313), indicating an even distribution of analgesic strategies across sexes and age brackets. Similarly, there was no significant variation in type of surgery across different analgesic groups (p = 0.964), suggesting that surgical intervention type did not drive analgesic selection.

**Table 5:** Comparison of demographic, clinical, and patient outcome variables by pain management strategy (N = 431).

Variables	Details	Pain management therapy				P Value
		Monotherapy	Opioid Based	Multimodal	Adjuvant Therapy	
Gender	Male	53	52	57	34	0.495
	Female	57	63	62	33	
	Not prefer to mention	5	4	10	1	
Age Group	19-28 Years	24	25	24	24	0.313
	29-38 Years	38	32	35	15	
	39-48 Years	28	30	31	18	
	49-58 Years	14	17	23	4	
	More than 59 Years	11	15	16	7	
Type Of Surgery	Post DCR (Dacryocystorhinostomy)	15	12	22	8	0.964
	Post Evisceration	8	13	13	5	
	Temporomandibular joint surgery and Impacted tooth Surgery	16	12	11	8	
	Post Repair of Corneal Laceration	9	16	16	13	
	Repair of Globe Rupture	13	11	11	7	
	Lid Laceration Repair	13	16	11	6	
	ORIF (Open Reduction and Internal Fixation) of Jaw Fractures	12	11	13	6	
	Maxillofacial Trauma Reconstruction	18	18	19	8	
	Others	11	10	13	7	
Patient Satisfaction	Very Dissatisfied	9	0	0	0	<0.001
	Dissatisfied	27	0	0	16	
	Neutral	57	27	13	36	
	Satisfied	22	58	63	16	
	Very Satisfied	0	34	53	0	
Side Effect	Nausea/Vomiting	30	32	19	0	<0.001
	Constipation	0	31	30	0	
	Sedation/Dizziness	0	19	0	13	
	GI Upset/Bleeding	22	0	28	17	
	Respiratory Issues	0	13	0	0	
	Hepatotoxicity	0	0	11	6	
	None	63	24	41	32	
Rescue Analgesia Used	Yes	34	33	30	26	0.172
	No	81	86	99	42	
Pain Interference In Function	No interference	0	60	44	0	<0.001
	Mild interference	31	30	55	25	
	Moderate interference	51	29	30	29	
	Severe interference	24	0	0	14	
	Complete inability	9	0	0	0	
Previous History Of Chronic Pain	Yes	34	29	36	19	0.838
	No	81	90	93	49	

In contrast, patient satisfaction was significantly associated with the type of pain management strategy (p < 0.001). The highest satisfaction rates were observed among recipients of multimodal therapy (53 patients reported being “very satisfied”) and opioid-based therapy (58 “satisfied” and 34 “very satisfied”). Notably, all patients who were very dissatisfied or dissatisfied received monotherapy or adjuvant therapy, with none reporting such dissatisfaction in the opioid-based or multimodal groups. A highly significant association was also observed between pain management strategy and side effects (p < 0.001). For example, sedation/dizziness and respiratory issues were exclusively reported in the opioid and adjuvant therapy groups, while constipation was absent in the monotherapy and adjuvant groups but prevalent in the opioid and multimodal groups. Interestingly, 63 monotherapy recipients reported no side effects, in contrast to only 24 opioid-based and 41 multimodal recipients.

The extent of pain interference with function was significantly linked to pain management modality (p < 0.001). All reports of severe interference and complete inability to function were concentrated in the monotherapy and adjuvant groups, while opioid-based and multimodal therapies were associated with greater reports of no interference (60 and 44 cases, respectively).

Although more patients receiving adjuvant therapy and monotherapy required rescue analgesia, the association was not statistically significant (p = 0.172). Similarly, previous history of chronic pain did not show a significant correlation with the analgesic strategy employed (p = 0.838).

### Correlation between pain management strategy, pain severity, and mobilization time

Table 6 displays the results of Pearson correlation analysis examining the relationships among the type of pain management administered, postoperative pain scores, and time to mobilization after surgery among the study participants (N = 431).

**Table 6:** Pearson correlation matrix between pain management type, postoperative pain score (NRS), and time to mobilization (N = 431).

Correlations				
		Type Of Pain Management	Postoperative Pain Score NRS	Type To Mobilization Postsurgery
Type Of Pain Management	Pearson Correlation	1	-.177^**^	-.116^*^
	Sig. (2-tailed)		.000	.016
	N	431	431	431
Postoperative Pain Score NRS	Pearson Correlation	-.177^**^	1	-.019
	Sig. (2-tailed)	.000		.691
	N	431	431	431
Type To Mobilization Postsurgery	Pearson Correlation	-.116^*^	-.019	1
	Sig. (2-tailed)	.016	.691	
	N	431	431	431

**Correlation is significant at the 0.01 level (2-tailed).

*Correlation is significant at the 0.05 level (2-tailed).

A statistically significant negative correlation was found between the type of pain management strategy and postoperative pain score (r = –0.177, *p* < 0.001), suggesting that patients receiving more advanced pain management approaches (such as multimodal or adjuvant therapies) experienced lower pain intensities. Additionally, a weaker but still significant negative correlation was observed between the type of pain management and time to mobilization post-surgery (r = –0.116, *p* = 0.016), indicating that better pain control was modestly associated with earlier postoperative mobilization.

No significant association was found between postoperative pain scores and time to mobilization (r = –0.019, *p* = 0.691), implying that while pain intensity and mobilization time are each related to the type of pain management used, they may not be directly interrelated in this sample.

### Analysis of variance for postoperative pain scores and mobilization time by type of pain management

Table 7 presents the results of one-way analysis of variance (ANOVA) assessing the impact of different pain management strategies on postoperative pain intensity and time to mobilization following surgery, with type of pain management as the independent variable.

**Table 7:** One-way ANOVA showing the effect of type of pain management on postoperative pain intensity and time to mobilization (N = 431).

ANOVA						
		Sum of Squares	df	Mean Square	F	Sig.
Postoperative Pain Score NRS	Between Groups	183.050	3	61.017	31.354	.000
	Within Groups	830.956	427	1.946		
	Total	1014.006	430			
Type To Mobilization Postsurgery	Between Groups	8181.481	3	2727.160	21.308	.000
	Within Groups	54649.559	427	127.985		
	Total	62831.041	430			

The ANOVA revealed a highly significant difference in postoperative pain scores (NRS) across the four pain management groups (F = 31.354, *p* < 0.001). This finding indicates that the choice of analgesic strategy had a substantial effect on patients’ reported pain intensity, with post hoc analysis (see Table 8) showing lower mean scores in multimodal and adjuvant therapy groups compared to monotherapy and opioid-based approaches.

**Table 8:** Tukey HSD post hoc comparisons of mean differences in pain score and mobilization time across pain management groups (N = 431).

Dependent Variable	(I) Type Of Pain Management	(J) Type Of Pain Management	Mean Difference (I-J)	Std. Error	Sig.	95% Confidence Interval	
						Lower Bound	Upper Bound
Postoperative Pain Score NRS	Monotherapy	Opioid-based	1.69255^*^	.18242	.000	1.2221	2.1630
		Multimodal	1.24803^*^	.17891	.000	.7866	1.7095
		Adjuvant therapy	.76797^*^	.21340	.002	.2176	1.3184
	Opioid-based	Monotherapy	-1.69255^*^	.18242	.000	-2.1630	-1.2221
		Multimodal	-.44452	.17731	.060	-.9018	.0128
		Adjuvant therapy	-.92458^*^	.21206	.000	-1.4715	-.3776
	Multimodal	Monotherapy	-1.24803^*^	.17891	.000	-1.7095	-.7866
		Opioid-based	.44452	.17731	.060	-.0128	.9018
		Adjuvant therapy	-.48006	.20905	.100	-1.0192	.0591
	Adjuvant therapy	Monotherapy	-.76797^*^	.21340	.002	-1.3184	-.2176
		Opioid-based	.92458^*^	.21206	.000	.3776	1.4715
		Multimodal	.48006	.20905	.100	-.0591	1.0192
Type To Mobilization Postsurgery	Monotherapy	Opioid-based	-6.57315^*^	1.47933	.000	-10.3886	-2.7577
		Multimodal	4.90296^*^	1.45088	.004	1.1609	8.6450
		Adjuvant therapy	-.51895	1.73062	.991	-4.9825	3.9446
	Opioid-based	Monotherapy	6.57315^*^	1.47933	.000	2.7577	10.3886
		Multimodal	11.47611^*^	1.43793	.000	7.7674	15.1848
		Adjuvant therapy	6.05420^*^	1.71978	.003	1.6186	10.4898
	Multimodal	Monotherapy	-4.90296^*^	1.45088	.004	-8.6450	-1.1609
		Opioid-based	-11.47611^*^	1.43793	.000	-15.1848	-7.7674
		Adjuvant therapy	-5.42191^*^	1.69536	.008	-9.7946	-1.0493
	Adjuvant therapy	Monotherapy	.51895	1.73062	.991	-3.9446	4.9825
		Opioid-based	-6.05420^*^	1.71978	.003	-10.4898	-1.6186
		Multimodal	5.42191^*^	1.69536	.008	1.0493	9.7946

*. The mean difference is significant at the 0.05 level.

Similarly, a statistically significant difference was observed in time to mobilization post-surgery across the pain management groups (F = 21.308, *p* < 0.001). This suggests that more effective analgesic protocols, particularly those incorporating multimodal or adjuvant components, were associated with earlier mobilization, potentially reflecting improved functional recovery and reduced pain interference.

These results underscore the clinical relevance of tailoring pain management strategies to optimize both subjective pain relief and objective recovery timelines in the postoperative setting.

### Post hoc Tukey HSD comparisons for pain score and mobilization time across pain management strategies

Table 8 presents the results of Tukey’s Honest Significant Difference (HSD) post hoc test, following a significant one-way ANOVA, to determine pairwise differences in postoperative pain scores and time to mobilization between different pain management strategies.

### Postoperative pain scores

Significant differences were observed between monotherapy and all other pain management strategies. Patients receiving monotherapy reported significantly higher pain scores than those receiving opioid-based (mean difference = 1.69, *p* < 0.001), multimodal (mean difference = 1.25, *p* < 0.001), and adjuvant therapies (mean difference = 0.77, *p* = 0.002). Similarly, patients receiving opioid-based regimens had higher pain scores than those treated with adjuvant therapy (mean difference = 0.92, *p* < 0.001). The difference between opioid-based and multimodal therapy was marginally non-significant (*p* = 0.060), while multimodal and adjuvant therapies did not significantly differ from each other (*p* = 0.100).

### Time to mobilization

There were also statistically significant differences in mobilization times across groups. Compared to monotherapy, patients in the opioid-based group had significantly delayed mobilization (mean difference = –6.57 hours, *p* < 0.001), while those in the multimodal group mobilized earlier (mean difference = 4.90 hours, *p* = 0.004). Multimodal therapy resulted in significantly faster mobilization compared to opioid-based therapy (mean difference = –11.48 hours, *p* < 0.001) and adjuvant therapy (mean difference = –5.42 hours, *p* = 0.008). Conversely, opioid-based therapy was associated with delayed mobilization relative to both multimodal and adjuvant strategies.

These findings emphasize that multimodal and adjuvant therapies are more effective in reducing pain and expediting recovery compared to monotherapy or opioid-only regimens.

### Analysis of variance for pain score and mobilization time by type of surgery

Table 9 presents the results of one-way ANOVA to evaluate whether the type of surgical procedure significantly influenced postoperative pain intensity and time to mobilization among the study participants (N = 431).

**Table 9:** One-way ANOVA showing the impact of type of surgery on postoperative pain and mobilization time (N = 431).

		Sum of Squares	df	Mean Square	F	Sig.
Postoperative Pain Score NRS	Between Groups	29.333	8	3.667	1.571	.131
	Within Groups	984.673	422	2.333		
	Total	1014.006	430			
Type To Mobilization Postsurgery	Between Groups	44772.245	8	5596.531	130.780	.000
	Within Groups	18058.796	422	42.793		
	Total	62831.041	430			

The analysis revealed that there was no statistically significant difference in postoperative pain scores across the nine types of surgical procedures (F = 1.571, *p* = 0.131). This suggests that regardless of surgical category—whether minimally invasive (e.g., DCR) or extensive (e.g., exenteration or trauma reconstruction)—the reported pain intensity on the Numerical Rating Scale (NRS) remained statistically similar.

In contrast, the type of surgery had a highly significant effect on the time to mobilization post-surgery (F = 130.780, *p* < 0.001). This indicates that patients undergoing more complex or invasive procedures, such as maxillofacial trauma reconstruction or globe rupture repair, experienced longer delays in mobilization, whereas patients with less invasive surgeries (e.g., lid laceration repair or DCR) mobilized earlier. The large F-value and highly significant p-value emphasize the strong influence of surgical complexity on postoperative recovery duration.

### Post hoc analysis of surgery type on pain and mobilization outcomes

Tukey’s HSD post hoc test was employed to examine pairwise differences in postoperative pain scores and time to mobilization across different surgical procedures (Table 10). The findings for postoperative pain (NRS) revealed that none of the pairwise comparisons between surgical types yielded statistically significant differences (all *p* > 0.05). This confirms the earlier ANOVA result suggesting that pain perception was not significantly influenced by the type of surgery.

**Table 10:** Tukey HSD post hoc comparisons of surgery types on postoperative pain and time to mobilization.

Multiple Comparisons							
Tukey HSD							
Dependent Variable	(I) Type Of Surgery	(J) Type Of Surgery	Mean Difference (I-J)	Std. Error	Sig.	95% Confidence Interval	
						Lower Bound	Upper Bound
Postoperative Pain Score NRS	Post DCR (Dacryocystorhinostomy)	Post Evisceration	.12942	.31744	1.000	-.8603	1.1192
		Temporomandibular joint surgery and Impacted tooth Surgery	-.02568	.30097	1.000	-.9641	.9127
		Post Repair of Corneal Laceration	-.14708	.29008	1.000	-1.0515	.7574
		Repair of Globe Rupture	-.62168	.31063	.543	-1.5902	.3469
		Lid Laceration Repair	-.53886	.30276	.696	-1.4828	.4051
		ORIF (Open Reduction and Internal Fixation) of Jaw Fractures	-.01692	.31063	1.000	-.9855	.9516
		Maxillofacial Trauma Reconstruction	-.33993	.27924	.952	-1.2106	.5307
		Others	-.59491	.31280	.613	-1.5702	.3804
	Post Evisceration	Post DCR (Dacryocystorhinostomy)	-.12942	.31744	1.000	-1.1192	.8603
		Temporomandibular joint surgery and Impacted tooth Surgery	-.15510	.33087	1.000	-1.1867	.8765
		Post Repair of Corneal Laceration	-.27650	.32100	.995	-1.2774	.7244
		Repair of Globe Rupture	-.75110	.33968	.400	-1.8102	.3080
		Lid Laceration Repair	-.66828	.33250	.537	-1.7050	.3684
		ORIF (Open Reduction and Internal Fixation) of Jaw Fractures	-.14634	.33968	1.000	-1.2055	.9128
		Maxillofacial Trauma Reconstruction	-.46935	.31123	.852	-1.4398	.5011
		Others	-.72433	.34167	.461	-1.7897	.3410
	Temporomandibular joint surgery and Impacted tooth Surgery	Post DCR (Dacryocystorhinostomy)	.02568	.30097	1.000	-.9127	.9641
		Post Evisceration	.15510	.33087	1.000	-.8765	1.1867
		Post Repair of Corneal Laceration	-.12139	.30472	1.000	-1.0715	.8287
		Repair of Globe Rupture	-.59600	.32435	.657	-1.6073	.4153
		Lid Laceration Repair	-.51318	.31681	.794	-1.5010	.4746
		ORIF (Open Reduction and Internal Fixation) of Jaw Fractures	.00876	.32435	1.000	-1.0025	1.0201
		Maxillofacial Trauma Reconstruction	-.31425	.29442	.978	-1.2322	.6037
		Others	-.56923	.32643	.719	-1.5870	.4486
	Post Repair of Corneal Laceration	Post DCR (Dacryocystorhinostomy)	.14708	.29008	1.000	-.7574	1.0515
		Post Evisceration	.27650	.32100	.995	-.7244	1.2774
		Temporomandibular joint surgery and Impacted tooth Surgery	.12139	.30472	1.000	-.8287	1.0715
		Repair of Globe Rupture	-.47460	.31427	.851	-1.4545	.5053
		Lid Laceration Repair	-.39179	.30649	.937	-1.3474	.5638
		ORIF (Open Reduction and Internal Fixation) of Jaw Fractures	.13016	.31427	1.000	-.8497	1.1100
		Maxillofacial Trauma Reconstruction	-.19286	.28328	.999	-1.0761	.6904
		Others	-.44783	.31642	.892	-1.4344	.5388
	Repair of Globe Rupture	Post DCR (Dacryocystorhinostomy)	.62168	.31063	.543	-.3469	1.5902
		Post Evisceration	.75110	.33968	.400	-.3080	1.8102
		Temporomandibular joint surgery and Impacted tooth Surgery	.59600	.32435	.657	-.4153	1.6073
		Post Repair of Corneal Laceration	.47460	.31427	.851	-.5053	1.4545
		Lid Laceration Repair	.08282	.32601	1.000	-.9337	1.0993
		ORIF (Open Reduction and Internal Fixation) of Jaw Fractures	.60476	.33333	.673	-.4346	1.6441
		Maxillofacial Trauma Reconstruction	.28175	.30429	.991	-.6670	1.2305
		Others	.02677	.33536	1.000	-1.0189	1.0724
	Lid Laceration Repair	Post DCR (Dacryocystorhinostomy)	.53886	.30276	.696	-.4051	1.4828
		Post Evisceration	.66828	.33250	.537	-.3684	1.7050
		Temporomandibular joint surgery and Impacted tooth Surgery	.51318	.31681	.794	-.4746	1.5010
		Post Repair of Corneal Laceration	.39179	.30649	.937	-.5638	1.3474
		Repair of Globe Rupture	-.08282	.32601	1.000	-1.0993	.9337
		ORIF (Open Reduction and Internal Fixation) of Jaw Fractures	.52195	.32601	.804	-.4945	1.5384
		Maxillofacial Trauma Reconstruction	.19893	.29625	.999	-.7248	1.1226
		Others	-.05604	.32808	1.000	-1.0790	.9669
	ORIF (Open Reduction and Internal Fixation) of Jaw Fractures	Post DCR (Dacryocystorhinostomy)	.01692	.31063	1.000	-.9516	.9855
		Post Evisceration	.14634	.33968	1.000	-.9128	1.2055
		Temporomandibular joint surgery and Impacted tooth Surgery	-.00876	.32435	1.000	-1.0201	1.0025
		Post Repair of Corneal Laceration	-.13016	.31427	1.000	-1.1100	.8497
		Repair of Globe Rupture	-.60476	.33333	.673	-1.6441	.4346
		Lid Laceration Repair	-.52195	.32601	.804	-1.5384	.4945
		Maxillofacial Trauma Reconstruction	-.32302	.30429	.979	-1.2718	.6258
		Others	-.57799	.33536	.732	-1.6236	.4677
	Maxillofacial Trauma Reconstruction	Post DCR (Dacryocystorhinostomy)	.33993	.27924	.952	-.5307	1.2106
		Post Evisceration	.46935	.31123	.852	-.5011	1.4398
		Temporomandibular joint surgery and Impacted tooth Surgery	.31425	.29442	.978	-.6037	1.2322
		Post Repair of Corneal Laceration	.19286	.28328	.999	-.6904	1.0761
		Repair of Globe Rupture	-.28175	.30429	.991	-1.2305	.6670
		Lid Laceration Repair	-.19893	.29625	.999	-1.1226	.7248
		ORIF (Open Reduction and Internal Fixation) of Jaw Fractures	.32302	.30429	.979	-.6258	1.2718
		Others	-.25497	.30651	.996	-1.2107	.7007
	Others	Post DCR (Dacryocystorhinostomy)	.59491	.31280	.613	-.3804	1.5702
		Post Evisceration	.72433	.34167	.461	-.3410	1.7897
		Temporomandibular joint surgery and Impacted tooth Surgery	.56923	.32643	.719	-.4486	1.5870
		Post Repair of Corneal Laceration	.44783	.31642	.892	-.5388	1.4344
		Repair of Globe Rupture	-.02677	.33536	1.000	-1.0724	1.0189
		Lid Laceration Repair	.05604	.32808	1.000	-.9669	1.0790
		ORIF (Open Reduction and Internal Fixation) of Jaw Fractures	.57799	.33536	.732	-.4677	1.6236
		Maxillofacial Trauma Reconstruction	.25497	.30651	.996	-.7007	1.2107
Type To Mobilization Postsurgery	Post DCR (Dacryocystorhinostomy)	Post Evisceration	-11.57922^*^	1.35942	.000	-15.8179	-7.3406
		Temporomandibular joint surgery and Impacted tooth Surgery	-24.39657^*^	1.28890	.000	-28.4153	-20.3778
		Post Repair of Corneal Laceration	-12.89133^*^	1.24227	.000	-16.7647	-9.0180
		Repair of Globe Rupture	-25.31805^*^	1.33028	.000	-29.4658	-21.1703
		Lid Laceration Repair	-2.55831	1.29655	.563	-6.6009	1.4843
		ORIF (Open Reduction and Internal Fixation) of Jaw Fractures	-13.48947^*^	1.33028	.000	-17.6373	-9.3417
		Maxillofacial Trauma Reconstruction	-25.84344^*^	1.19583	.000	-29.5720	-22.1149
		Others	.22679	1.33959	1.000	-3.9500	4.4036
	Post Evisceration	Post DCR (Dacryocystorhinostomy)	11.57922^*^	1.35942	.000	7.3406	15.8179
		Temporomandibular joint surgery and Impacted tooth Surgery	-12.81735^*^	1.41696	.000	-17.2354	-8.3993
		Post Repair of Corneal Laceration	-1.31211	1.37468	.990	-5.5983	2.9741
		Repair of Globe Rupture	-13.73883^*^	1.45470	.000	-18.2745	-9.2031
		Lid Laceration Repair	9.02090^*^	1.42392	.000	4.5812	13.4607
		ORIF (Open Reduction and Internal Fixation) of Jaw Fractures	-1.91026	1.45470	.927	-6.4460	2.6255
		Maxillofacial Trauma Reconstruction	-14.26422^*^	1.33286	.000	-18.4201	-10.1084
		Others	11.80600^*^	1.46322	.000	7.2437	16.3683
	Temporomandibular joint surgery and Impacted tooth Surgery	Post DCR (Dacryocystorhinostomy)	24.39657^*^	1.28890	.000	20.3778	28.4153
		Post Evisceration	12.81735^*^	1.41696	.000	8.3993	17.2354
		Post Repair of Corneal Laceration	11.50524^*^	1.30498	.000	7.4364	15.5741
		Repair of Globe Rupture	-.92148	1.38902	.999	-5.2524	3.4095
		Lid Laceration Repair	21.83825^*^	1.35676	.000	17.6079	26.0686
		ORIF (Open Reduction and Internal Fixation) of Jaw Fractures	10.90709^*^	1.38902	.000	6.5762	15.2380
		Maxillofacial Trauma Reconstruction	-1.44688	1.26086	.966	-5.3782	2.4844
		Others	24.62335^*^	1.39794	.000	20.2646	28.9821
	Post Repair of Corneal Laceration	Post DCR (Dacryocystorhinostomy)	12.89133^*^	1.24227	.000	9.0180	16.7647
		Post Evisceration	1.31211	1.37468	.990	-2.9741	5.5983
		Temporomandibular joint surgery and Impacted tooth Surgery	-11.50524^*^	1.30498	.000	-15.5741	-7.4364
		Repair of Globe Rupture	-12.42672^*^	1.34587	.000	-16.6231	-8.2303
		Lid Laceration Repair	10.33301^*^	1.31254	.000	6.2405	14.4255
		ORIF (Open Reduction and Internal Fixation) of Jaw Fractures	-.59815	1.34587	1.000	-4.7945	3.5982
		Maxillofacial Trauma Reconstruction	-12.95212^*^	1.21315	.000	-16.7347	-9.1696
		Others	13.11811^*^	1.35507	.000	8.8930	17.3432
	Repair of Globe Rupture	Post DCR (Dacryocystorhinostomy)	25.31805^*^	1.33028	.000	21.1703	29.4658
		Post Evisceration	13.73883^*^	1.45470	.000	9.2031	18.2745
		Temporomandibular joint surgery and Impacted tooth Surgery	.92148	1.38902	.999	-3.4095	5.2524
		Post Repair of Corneal Laceration	12.42672^*^	1.34587	.000	8.2303	16.6231
		Lid Laceration Repair	22.75973^*^	1.39613	.000	18.4066	27.1128
		ORIF (Open Reduction and Internal Fixation) of Jaw Fractures	11.82857^*^	1.42751	.000	7.3776	16.2795
		Maxillofacial Trauma Reconstruction	-.52540	1.30313	1.000	-4.5885	3.5377
		Others	25.54483^*^	1.43619	.000	21.0668	30.0228
	Lid Laceration Repair	Post DCR (Dacryocystorhinostomy)	2.55831	1.29655	.563	-1.4843	6.6009
		Post Evisceration	-9.02090^*^	1.42392	.000	-13.4607	-4.5812
		Temporomandibular joint surgery and Impacted tooth Surgery	-21.83825^*^	1.35676	.000	-26.0686	-17.6079
		Post Repair of Corneal Laceration	-10.33301^*^	1.31254	.000	-14.4255	-6.2405
		Repair of Globe Rupture	-22.75973^*^	1.39613	.000	-27.1128	-18.4066
		ORIF (Open Reduction and Internal Fixation) of Jaw Fractures	-10.93116^*^	1.39613	.000	-15.2843	-6.5781
		Maxillofacial Trauma Reconstruction	-23.28513^*^	1.26868	.000	-27.2408	-19.3294
		Others	2.78510	1.40500	.557	-1.5957	7.1659
	ORIF (Open Reduction and Internal Fixation) of Jaw Fractures	Post DCR (Dacryocystorhinostomy)	13.48947^*^	1.33028	.000	9.3417	17.6373
		Post Evisceration	1.91026	1.45470	.927	-2.6255	6.4460
		Temporomandibular joint surgery and Impacted tooth Surgery	-10.90709^*^	1.38902	.000	-15.2380	-6.5762
		Post Repair of Corneal Laceration	.59815	1.34587	1.000	-3.5982	4.7945
		Repair of Globe Rupture	-11.82857^*^	1.42751	.000	-16.2795	-7.3776
		Lid Laceration Repair	10.93116^*^	1.39613	.000	6.5781	15.2843
		Maxillofacial Trauma Reconstruction	-12.35397^*^	1.30313	.000	-16.4171	-8.2908
		Others	13.71626^*^	1.43619	.000	9.2383	18.1942
	Maxillofacial Trauma Reconstruction	Post DCR (Dacryocystorhinostomy)	25.84344^*^	1.19583	.000	22.1149	29.5720
		Post Evisceration	14.26422^*^	1.33286	.000	10.1084	18.4201
		Temporomandibular joint surgery and Impacted tooth Surgery	1.44688	1.26086	.966	-2.4844	5.3782
		Post Repair of Corneal Laceration	12.95212^*^	1.21315	.000	9.1696	16.7347
		Repair of Globe Rupture	.52540	1.30313	1.000	-3.5377	4.5885
		Lid Laceration Repair	23.28513^*^	1.26868	.000	19.3294	27.2408
		ORIF (Open Reduction and Internal Fixation) of Jaw Fractures	12.35397^*^	1.30313	.000	8.2908	16.4171
		Others	26.07023^*^	1.31263	.000	21.9775	30.1630
	Others	Post DCR (Dacryocystorhinostomy)	-.22679	1.33959	1.000	-4.4036	3.9500
		Post Evisceration	-11.80600^*^	1.46322	.000	-16.3683	-7.2437
		Temporomandibular joint surgery and Impacted tooth Surgery	-24.62335^*^	1.39794	.000	-28.9821	-20.2646
		Post Repair of Corneal Laceration	-13.11811^*^	1.35507	.000	-17.3432	-8.8930
		Repair of Globe Rupture	-25.54483^*^	1.43619	.000	-30.0228	-21.0668
		Lid Laceration Repair	-2.78510	1.40500	.557	-7.1659	1.5957
		ORIF (Open Reduction and Internal Fixation) of Jaw Fractures	-13.71626^*^	1.43619	.000	-18.1942	-9.2383
		Maxillofacial Trauma Reconstruction	-26.07023^*^	1.31263	.000	-30.1630	-21.9775

*. The mean difference is significant at the 0.05 level.

In contrast, time to mobilization post-surgery showed numerous statistically significant differences across surgical categories (*p* < 0.001). Patients undergoing Temporomandibular joint surgery and Impacted tooth Surgery, Repair of Globe Rupture, Maxillofacial Trauma Reconstruction, and ORIF of jaw fractures had significantly longer mobilization times compared to those who had less invasive procedures like DCR, Lid Laceration Repair, or Post Evisceration. For example, patients who underwent Maxillofacial Trauma Reconstruction took on average 25.84 hours longer to mobilize than those who had a DCR procedure (*p* < 0.001, 95% CI: 22.11–29.57).

Conversely, surgeries such as Lid Laceration Repair and ‘Others’ exhibited significantly shorter mobilization durations when compared with more extensive surgeries. For instance, the difference in mobilization time between Lid Laceration Repair and Temporomandibular joint surgery and Impacted tooth Surgery was −21.83 hours (*p* < 0.001), highlighting how surgical invasiveness influences recovery dynamics.

The Independent Samples Test was conducted to compare postoperative pain scores (measured by the Numerical Rating Scale, NRS) and time to mobilization between male and female participants. Levene’s Test for Equality of Variances indicated no significant difference in variances for postoperative pain scores (*F* = 1.767, *p* = 0.184) or mobilization time (*F* = 3.705, *p* = 0.055), supporting the assumption of homogeneity. The t-test results revealed no statistically significant gender differences in postoperative pain scores (*t* = 1.717, *p* = 0.087) or mobilization time (*t* = -0.488, *p* = 0.626). The mean differences (0.26 for pain scores and -0.59 hours for mobilization time) and their 95% confidence intervals further confirmed the lack of significant gender-based disparities in these outcomes (Table 11).

**Table 11:** Independent samples test comparing postoperative pain scores and mobilization time by gender.

		Levene’s Test for Equality of Variances		t-test for Equality of Means						
		F	Sig.	t	df	Sig. (2-tailed)	Mean Difference	Std. Error Difference	95% Confidence Interval of the Difference	
									Lower	Upper
Postoperative Pain Score NRS	Equal variances assumed	1.767	.184	1.717	409	.087	.25746	.14994	-.03729	.55221
	Equal variances not assumed			1.725	408.989	.085	.25746	.14926	-.03596	.55088
Type To Mobilization Postsurgery	Equal variances assumed	3.705	.055	-.488	409	.626	-.58544	1.19877	-2.94195	1.77107
	Equal variances not assumed			-.490	408.963	.624	-.58544	1.19416	-2.93290	1.76202

Table 12 presents the results of a one-way ANOVA examining whether the frequency of analgesia use is associated with differences in postoperative pain scores (measured by the Numerical Rating Scale, NRS) and the time to mobilization after surgery among 431 patients. The analysis for postoperative pain score (NRS) showed no statistically significant difference between groups with different frequencies of analgesia use (F = 1.422,p = 0.236*F* = 1.422,*p* = 0.236), indicating that the frequency of analgesia did not significantly impact reported pain intensity. In contrast, the ANOVA for time to mobilization postsurgery revealed a statistically significant difference between groups (F = 3.447,p = 0.017*F* = 3.447,*p* = 0.017), suggesting that the frequency of analgesia use is associated with variations in the time it takes for patients to mobilize after surgery. This finding highlights the potential influence of analgesia management on postoperative recovery dynamics.

**Table 12:** One-way ANOVA showing the association between frequency of analgesia use, postoperative pain score, and time to mobilization.

ANOVA						
		Sum of Squares	df	Mean Square	F	Sig.
Postoperative_Pain_Score_NRS	Between Groups	10.030	3	3.343	1.422	.236
	Within Groups	1003.976	427	2.351		
	Total	1014.006	430			
Type_To_Mobilization_Postsurgery	Between Groups	1485.610	3	495.203	3.447	.017
	Within Groups	61345.430	427	143.666		
	Total	62831.041	430			

Table 13 displays the results of a one-way ANOVA evaluating whether patient satisfaction with pain management is associated with differences in postoperative pain scores (NRS) and time to mobilization after surgery among 431 participants. The analysis found a statistically significant difference in postoperative pain scores across different levels of pain satisfaction (F = 8.717,p < 0.001*F* = 8.717,*p* < 0.001), indicating that satisfaction with pain management is closely linked to the intensity of pain reported after surgery. However, there was no significant difference in time to mobilization between groups with varying pain satisfaction (F = 0.111,p = 0.979*F* = 0.111,*p* = 0.979), suggesting that satisfaction with pain control did not influence how quickly patients were able to mobilize postoperatively.

**Table 13:** One-way ANOVA showing the association between pain satisfaction, postoperative pain score, and time to mobilization.

		Sum of Squares	df	Mean Square	F	Sig.
Postoperative_Pain_Score_NRS	Between Groups	76.715	4	19.179	8.717	.000
	Within Groups	937.291	426	2.200		
	Total	1014.006	430			
Type_To_Mobilization_Postsurgery	Between Groups	65.200	4	16.300	.111	.979
	Within Groups	62765.840	426	147.338		
	Total	62831.041	430			

Table 14 presents the results of Tukey HSD post hoc tests comparing postoperative pain scores and time to mobilization among different patient satisfaction groups. For postoperative pain scores (NRS), several significant differences were observed. Patients who were “Very Dissatisfied” reported significantly higher pain scores compared to those who were “Satisfied” (mean difference = 1.41, p = 0.046) and “Very Satisfied” (mean difference = 1.71, p = 0.010). Similarly, “Dissatisfied” patients had higher pain scores than both “Satisfied” (mean difference = 1.02, p = 0.001) and “Very Satisfied” (mean difference = 1.32, p < 0.001). “Neutral” patients also reported higher pain scores than “Very Satisfied” patients (mean difference = 0.72, p = 0.004). These findings indicate that greater satisfaction with pain management is associated with lower reported postoperative pain.

**Table 14:** Tukey HSD multiple comparisons of patient satisfaction groups on postoperative pain score and time to mobilization (N = 431).

Dependent Variable	(I) Patient Satisfaction	(J) Patient Satisfaction	Mean Difference (I-J)	Std. Error	Sig.	95% Confidence Interval	
						Lower Bound	Upper Bound
Postoperative Pain Score NRS	Very Dissatisfied	Dissatisfied	.38501	.54372	.955	-1.1045	1.8745
		Neutral	.98613	.51089	.303	-.4135	2.3857
		Satisfied	1.40587^*^	.50824	.046	.0136	2.7982
		Very Satisfied	1.70728^*^	.51938	.010	.2844	3.1301
	Dissatisfied	Very Dissatisfied	-.38501	.54372	.955	-1.8745	1.1045
		Neutral	.60112	.26021	.144	-.1117	1.3140
		Satisfied	1.02086^*^	.25496	.001	.3224	1.7193
		Very Satisfied	1.32227^*^	.27651	.000	.5648	2.0798
	Neutral	Very Dissatisfied	-.98613	.51089	.303	-2.3857	.4135
		Dissatisfied	-.60112	.26021	.144	-1.3140	.1117
		Satisfied	.41974	.17430	.115	-.0578	.8972
		Very Satisfied	.72115^*^	.20453	.004	.1608	1.2815
	Satisfied	Very Dissatisfied	-1.40587^*^	.50824	.046	-2.7982	-.0136
		Dissatisfied	-1.02086^*^	.25496	.001	-1.7193	-.3224
		Neutral	-.41974	.17430	.115	-.8972	.0578
		Very Satisfied	.30141	.19781	.548	-.2405	.8433
	Very Satisfied	Very Dissatisfied	-1.70728^*^	.51938	.010	-3.1301	-.2844
		Dissatisfied	-1.32227^*^	.27651	.000	-2.0798	-.5648
		Neutral	-.72115^*^	.20453	.004	-1.2815	-.1608
		Satisfied	-.30141	.19781	.548	-.8433	.2405
Type To Mobilization Postsurgery	Very Dissatisfied	Dissatisfied	2.49561	4.44942	.981	-9.6936	14.6848
		Neutral	2.75422	4.18075	.965	-8.6989	14.2074
		Satisfied	2.61866	4.15903	.970	-8.7750	14.0123
		Very Satisfied	2.48467	4.25022	.977	-9.1588	14.1281
	Dissatisfied	Very Dissatisfied	-2.49561	4.44942	.981	-14.6848	9.6936
		Neutral	.25861	2.12938	1.000	-5.5748	6.0920
		Satisfied	.12305	2.08641	1.000	-5.5927	5.8388
		Very Satisfied	-.01093	2.26274	1.000	-6.2097	6.1878
	Neutral	Very Dissatisfied	-2.75422	4.18075	.965	-14.2074	8.6989
		Dissatisfied	-.25861	2.12938	1.000	-6.0920	5.5748
		Satisfied	-.13556	1.42634	1.000	-4.0430	3.7719
		Very Satisfied	-.26954	1.67372	1.000	-4.8547	4.3156
	Satisfied	Very Dissatisfied	-2.61866	4.15903	.970	-14.0123	8.7750
		Dissatisfied	-.12305	2.08641	1.000	-5.8388	5.5927
		Neutral	.13556	1.42634	1.000	-3.7719	4.0430
		Very Satisfied	-.13398	1.61870	1.000	-4.5684	4.3004
	Very Satisfied	Very Dissatisfied	-2.48467	4.25022	.977	-14.1281	9.1588
		Dissatisfied	.01093	2.26274	1.000	-6.1878	6.2097
		Neutral	.26954	1.67372	1.000	-4.3156	4.8547
		Satisfied	.13398	1.61870	1.000	-4.3004	4.5684

*. The mean difference is significant at the 0.05 level.

In contrast, no significant differences were found between satisfaction groups for time to mobilization after surgery, as all pairwise comparisons yielded non-significant results (all p > 0.05). This suggests that patient satisfaction with pain management did not influence the speed of postoperative mobilization.

Table 15 presents the results of independent samples t-tests evaluating whether a previous history of chronic pain is associated with differences in postoperative pain scores (NRS) and time to mobilization after surgery. Levene’s test for equality of variances was not significant for either outcome (pain score: F = 0.025,p = 0.875*F* = 0.025,*p* = 0.875; mobilization time: F = 2.224,p = 0.137*F* = 2.224,*p* = 0.137), indicating that the assumption of equal variances holds.

**Table 15:** Independent samples t-test comparing postoperative pain score and time to mobilization by previous history of chronic pain (N = 431).

		Levene’s Test for Equality of Variances		t-test for Equality of Means						
		F	Sig.	t	df	Sig. (2-tailed)	Mean Difference	Std. Error Difference	95% Confidence Interval of the Difference	
									Lower	Upper
Type_To_Mobilization_Postsurgery	Equal variances assumed	2.224	.137	2.984	429	.003	3.86144	1.29396	1.31814	6.40474
	Equal variances not assumed			3.141	234.166	.002	3.86144	1.22941	1.43931	6.28356
Postoperative_Pain_Score_NRS	Equal variances assumed	.025	.875	2.454	429	.015	.40474	.16493	.08057	.72890
	Equal variances not assumed			2.511	220.560	.013	.40474	.16119	.08706	.72242

The t-test revealed that patients with a previous history of chronic pain reported significantly higher postoperative pain scores compared to those without such a history (t = 2.454,df = 429,p = 0.015*t* = 2.454,*df* = 429,*p* = 0.015; mean difference = 0.40, 95% CI: 0.08 to 0.73). Additionally, these patients experienced a significantly longer time to mobilization after surgery (t = 2.984,df = 429,p = 0.003*t* = 2.984,*df* = 429,*p* = 0.003; mean difference = 3.86 hours, 95% CI: 1.32 to 6.40). These findings suggest that a history of chronic pain is associated with both greater postoperative pain and delayed recovery in terms of mobilization.

## Discussion

This study addressed the primary research question of whether postoperative pain management strategies are associated with differences in pain intensity and mobilization time. Our findings suggest that multimodal therapy was associated with lower pain scores and earlier mobilization compared to other approaches, while secondary analyses highlighted differences in satisfaction and side-effect profiles. Postoperative pain management remains a critical determinant of surgical recovery, profoundly impacting patient outcomes, healthcare utilization, and long-term quality of life. Our study from a Pakistani tertiary care center provides robust evidence supporting the superior efficacy of multimodal analgesia over traditional opioid-centric approaches, contributing to the growing global consensus on optimized perioperative pain management while highlighting unique considerations for resource-limited settings. The results demonstrate that multimodal analgesia—combining opioids with non-opioid adjuvants—achieved significantly better outcomes than monotherapy or opioid-only regimens. Patients receiving multimodal therapy reported 33% lower pain scores (mean NRS 3.39 vs. 5.08; *p* < 0.001) and mobilized nearly 7 hours earlier (34.6 vs. 41.2 hours; *p* < 0.001) compared to opioid-based groups. These findings align with multiple international studies, including the ERAS Society guidelines [14,15], which recommend multimodal approaches to reduce opioid consumption by 30–50% while improving pain control. A 2023 multinational cohort study [16,17] further demonstrated similar benefits across diverse healthcare systems, particularly in lower-resource settings. Mechanistic evidence from basic science research [18–20] supports these clinical observations through the concept of “analgesic synergy,” where combining agents targeting different pain pathways (e.g., NSAIDs for inflammatory pain, gabapentinoids for neuropathic components) produces enhanced therapeutic effects. Our findings indicate that multimodal therapy provides superior outcomes compared to monotherapy and adjuvant strategies. Compared to opioid-based regimens, multimodal therapy was associated with significantly earlier mobilization, although the reduction in pain scores showed only a non-significant trend (p = 0.060). This highlights that while multimodal therapy may offer functional advantages, the clinical difference in pain intensity relative to opioid-based regimens may be modest.

The opioid-sparing benefits observed in our study are particularly noteworthy, with 27.6% of patients receiving opioid-only regimens experiencing significantly more side effects (nausea 18.8%, constipation 14.2%) compared to multimodal groups (*p* < 0.001). These findings reinforce the 2022 CDC recommendations [21] advocating multimodal approaches as first-line therapy to mitigate opioid-related complications. Large pharmacovigilance studies [22,23] corroborate our results, showing opioid monotherapy is associated with a 2–3 × higher risk of gastrointestinal and respiratory complications. Interestingly, our Pakistani cohort exhibited higher opioid tolerance than Western populations in the PAIN-OUT registry [24], suggesting potential genetic or cultural influences on pain perception and medication response that warrant further investigation.

Psychological and gender-specific factors also emerged as critical determinants of pain outcomes. The significant association between preoperative anxiety/depression (29.9% prevalence) and increased pain perception (*p* = 0.005) aligns with the PROTECT study [25,26], which established psychological distress as predicting 23% of postoperative pain variance. These findings support the integration of psychological screening into preoperative assessments, particularly given the demonstrated efficacy of targeted interventions in high-risk patients [27,28]. Gender differences in rescue analgesia use further highlight the importance of sex-specific pain management strategies, as evidenced by NIH-funded research [28,29] on differential pain processing and opioid metabolism.

Our analysis of surgical outcomes revealed a notable dissociation between pain scores and functional recovery. While pain scores did not significantly differ across procedure types (*p* = 0.131), mobilization times varied markedly (*p* < 0.001), with more invasive surgeries such as maxillofacial trauma repairs showing the longest recovery periods. This observation supports the 2023 ESPEN guidelines [30–32], which emphasize functional metrics over pain scores as more meaningful endpoints for evaluating postoperative recovery. Recent work on inflammatory biomarkers [11] further elucidates this phenomenon, identifying IL-6 trajectories as superior predictors of functional recovery compared to subjective pain reports.

Despite its contributions, our study has limitations common to LMIC research, including its single-center design and lack of long-term follow-up [33]. Future studies should address these gaps by incorporating precision medicine approaches, such as pharmacogenomic testing for opioid metabolism (e.g., CYP2D6 variants) [34,35], and leveraging digital health technologies for remote pain monitoring [36]. Cost-effectiveness analyses, similar to those in the VALUE study [37], are also needed to evaluate the economic feasibility of multimodal regimens in resource-limited settings.

From a clinical and policy perspective, our findings support three key actions: (1) adapting evidence-based, procedure-specific guidelines (e.g., PROSPECT) to local formularies; (2) implementing WHO-endorsed pain management training for healthcare providers in LMICs; and (3) prioritizing research on sustainable multimodal strategies, as outlined by the IASP Global Task Force [38]. By integrating our findings with global best practices, healthcare systems can develop tailored, effective, and safer pain management protocols that optimize both clinical outcomes and patient experiences in diverse settings. Our use of **mutually exclusive, operationally defined exposure groups** with explicit timing windows and a hierarchy reduces misclassification and enhances interpretability of observed **associations** between analgesic strategies and outcomes in this cross-sectional sample.

## Strengths and limitations

One of the strengths of this study is the large sample size (n = 431) and the comprehensive inclusion of various surgery types and pain regimens. Moreover, the use of validated tools like the NRS for pain scoring enhances the reliability of findings.

This study has several limitations that should be considered when interpreting the findings. First, pain intensity was assessed using patient-reported scores, which are inherently subjective and subject to recall bias, particularly when a single score was expected to summarize pain over multiple days. Second, the classification of pain management strategies did not account for rescue analgesia beyond recording its use as a separate variable. Although this approach preserved comparability across primary groups, it may have broadened the effective administration route (e.g., oral monotherapy later supplemented with intravenous opioids), introducing heterogeneity not fully captured in our analysis. Third, differences in dosing regimens and routes of drug administration (oral, intravenous, intramuscular, or regional) may have influenced pharmacological efficacy and thus affected outcomes.

Fourth, the interpretation of mean pain scores and mobilisation times should be viewed cautiously, as these measures were likely influenced by multiple confounding factors, including surgical complexity, baseline functional status, preoperative anxiety or depression, comorbid conditions, and nutritional or psychological status. Although we applied appropriate statistical analyses, residual confounding cannot be excluded. Fifth, adverse events were collected descriptively and should not be interpreted as causal effects of specific drug classes, given the frequent use of multimodal regimens and heterogeneous dosing. Finally, hepatotoxicity was documented from routine clinician records rather than standardized liver function testing, and therefore represents exploratory rather than definitive evidence.

Taken together, these limitations indicate that while our results highlight meaningful trends in favor of multimodal analgesia, causal inferences should be made with caution. Future randomized and longitudinal studies with standardized outcome assessments are needed to overcome these methodological constraints and confirm the robustness of our findings.

## Conclusion

This study highlights the critical role of effective postoperative pain management in improving patient outcomes and functional recovery. Multimodal pain management improves postoperative outcomes, particularly functional recovery through earlier mobilization. Although pain scores were lower compared to opioid-based therapy, the difference did not reach statistical significance, underscoring the need for larger, more powered studies to confirm these findings. The findings support the integration of multimodal and individualized pain management plans, especially in surgical settings with diverse patient needs.

Furthermore, gender differences, psychological states such as preoperative anxiety or depression, and type of surgery were found to influence pain perception and recovery, emphasizing the need for a holistic and patient-centered approach. Policymakers and healthcare providers should prioritize standardized protocols that promote the judicious use of opioids while enhancing access to multimodal analgesics and supportive therapies.

Future multicenter, longitudinal studies are warranted to validate these findings and further optimize pain management protocols across varied clinical settings.

## Supporting information

S1 Table Agent-level analgesic exposures by mutually exclusive group, with doses, routes, timing, and rescue use. (DOCX)

S2 Table Agent-level exposure details by analgesic strategy (N = 431). (DOCX)

S3 Table Route-appropriate equianalgesic conversion factors to morphine milligram equivalents (MME), and computation examples. (DOCX)

## References

[1] Gan TJ. Poorly controlled postoperative pain: prevalence, consequences, and prevention. J Pain Res. 2017;10:2287–98. doi: 10.2147/JPR.S144066 29026331 PMC5626380

[2] Gan TJ, Epstein RS, Leone-Perkins ML, Salimi T, Iqbal SU, Whang PG. Practice Patterns and Treatment Challenges in Acute Postoperative Pain Management: A Survey of Practicing Physicians. Pain Ther. 2018;7(2):205–16. doi: 10.1007/s40122-018-0106-9 30367388 PMC6251830

[3] Fletcher D, Lavand’homme P. Towards better predictive models of chronic post-surgical pain: fitting to the dynamic nature of the pain itself. Br J Anaesth. 2022;129(3):281–4. doi: 10.1016/j.bja.2022.06.010 35835605

[4] Hah JM, Cramer E, Hilmoe H, Schmidt P, McCue R, Trafton J, et al. Factors Associated With Acute Pain Estimation, Postoperative Pain Resolution, Opioid Cessation, and Recovery: Secondary Analysis of a Randomized Clinical Trial. JAMA Netw Open. 2019;2(3):e190168. doi: 10.1001/jamanetworkopen.2019.0168 30821824 PMC6484627

[5] Wang J, Doan LV. Clinical pain management: Current practice and recent innovations in research. Cell Rep Med. 2024;5(10):101786. doi: 10.1016/j.xcrm.2024.101786 39383871 PMC11513809

[6] Sun M, Wang X, Lu Z, Yang Y, Lv S, Miao M, et al. Chronic Postsurgical Pain Raises Risk of Dementia. Eur J Pain. 2025;29(4):e70002. doi: 10.1002/ejp.70002 39981810

[7] Sun H-L, Bai W, Chen P, Zhang L, Smith RD, Su Z, et al. Pain trajectories and their associations with cognition among older adults: a 10-year cohort study from network perspective. Age Ageing. 2024;53(3):afae054. doi: 10.1093/ageing/afae054 38521972 PMC10960922

[8] Morriss WW, Roques CJ. Pain management in low- and middle-income countries. BJA Educ. 2018;18(9):265–70. doi: 10.1016/j.bjae.2018.05.006 33456843 PMC7807826

[9] Peeler A, Afolabi O, Adcock M, Evans C, Nkhoma K, van Breevoort D, Farrant L, Harding R: Primary palliative care in low- and middle-income countries: A systematic review and thematic synthesis of the evidence for models and outcomes. Palliat Med. 2024;38(8):776–89. 38693716 10.1177/02692163241248324 PMC11487876

[10] Haque G, Asif F, Ahmed FA, Ayub F, Syed SUH, Pradhan NA, et al. Assessment of Patient Safety in a Low-Resource Health Care System: Proposal for a Multimethod Study. JMIR Res Protoc. 2024;13:e50532. doi: 10.2196/50532 38536223 PMC11007612

[11] Shi Y, Wu W. Multimodal non-invasive non-pharmacological therapies for chronic pain: mechanisms and progress. BMC Med. 2023;21(1):372. doi: 10.1186/s12916-023-03076-2 37775758 PMC10542257

[12] Helander EM, Menard BL, Harmon CM, Homra BK, Allain AV, Bordelon GJ, et al. Multimodal Analgesia, Current Concepts, and Acute Pain Considerations. Curr Pain Headache Rep. 2017;21(1):3. doi: 10.1007/s11916-017-0607-y 28132136

[13] Schwenk ES, Mariano ER. Designing the ideal perioperative pain management plan starts with multimodal analgesia. Korean J Anesthesiol. 2018;71(5):345–52. doi: 10.4097/kja.d.18.00217 30139215 PMC6193589

[14] Nelson G, Fotopoulou C, Taylor J, Glaser G, Bakkum-Gamez J, Meyer LA, et al. Enhanced recovery after surgery (ERAS®) society guidelines for gynecologic oncology: Addressing implementation challenges - 2023 update. Gynecol Oncol. 2023;173:58–67. doi: 10.1016/j.ygyno.2023.04.009 37086524

[15] Gustafsson UO, Scott MJ, Hubner M, Nygren J, Demartines N, Francis N, et al. Guidelines for Perioperative Care in Elective Colorectal Surgery: Enhanced Recovery After Surgery (ERAS®) Society Recommendations: 2018. World J Surg. 2019;43(3):659–95. doi: 10.1007/s00268-018-4844-y 30426190

[16] Moreira RO, Valerio CM, Villela-Nogueira CA, Cercato C, Gerchman F, Lottenberg AMP, et al. Brazilian evidence-based guideline for screening, diagnosis, treatment, and follow-up of metabolic dysfunction-associated steatotic liver disease (MASLD) in adult individuals with overweight or obesity: A joint position statement from the Brazilian Society of Endocrinology and Metabolism (SBEM), Brazilian Society of Hepatology (SBH), and Brazilian Association for the Study of Obesity and Metabolic Syndrome (Abeso). Arch Endocrinol Metab. 2023;67(6):e230123. 10.20945/2359-4292-2023-0123 38048417

[17] Vij S, Too A, Tsang V, Kreutzwiser D. Analgesic medication considerations for chronic pain management post-bariatric surgery. Expert Opin Drug Metab Toxicol. 2024;20(10):967–76. doi: 10.1080/17425255.2024.2398631 39193986

[18] Smith PA. Neuropathic pain; what we know and what we should do about it. Front Pain Res (Lausanne). 2023;4:1220034. doi: 10.3389/fpain.2023.1220034 37810432 PMC10559888

[19] Cao B, Xu Q, Shi Y, Zhao R, Li H, Zheng J, et al. Pathology of pain and its implications for therapeutic interventions. Signal Transduct Target Ther. 2024;9(1):155. doi: 10.1038/s41392-024-01845-w 38851750 PMC11162504

[20] Portaccio E, Magyari M, Havrdova EK, Ruet A, Brochet B, Scalfari A, et al. Multiple sclerosis: emerging epidemiological trends and redefining the clinical course. Lancet Reg Health Eur. 2024;44:100977. doi: 10.1016/j.lanepe.2024.100977 39444703 PMC11496978

[21] Dowell D, Ragan KR, Jones CM, Baldwin GT, Chou R. CDC Clinical Practice Guideline for Prescribing Opioids for Pain - United States, 2022. MMWR Recomm Rep. 2022;71(3):1–95. doi: 10.15585/mmwr.rr7103a1 36327391 PMC9639433

[22] Boncyk C, Rolfsen ML, Richards D, Stollings JL, Mart MF, Hughes CG, et al. Management of pain and sedation in the intensive care unit. BMJ. 2024;387:e079789. doi: 10.1136/bmj-2024-079789 39653416

[23] Jeddi HM, Busse JW, Sadeghirad B, Levine M, Zoratti MJ, Wang L, et al. Cannabis for medical use versus opioids for chronic non-cancer pain: a systematic review and network meta-analysis of randomised clinical trials. BMJ Open. 2024;14(1):e068182. doi: 10.1136/bmjopen-2022-068182 38171632 PMC10773353

[24] Amjad MA, Siddiqui AM, Bashir K, Ghafoor A-U-R, Durrani RS. Prevalence of chronic pain in Pakistan - a national survey. J Pak Med Assoc. 2023;73(6):1217–20. doi: 10.47391/JPMA.6671 37427618

[25] Hinrichs-Rocker A, Schulz K, Järvinen I, Lefering R, Simanski C, Neugebauer EAM. Psychosocial predictors and correlates for chronic post-surgical pain (CPSP) - a systematic review. Eur J Pain. 2009;13(7):719–30. doi: 10.1016/j.ejpain.2008.07.015 18952472

[26] Einhorn LM, Krishnan P, Poirier C, Ingelmo P. Chronic Postsurgical Pain in Children and Adolescents: A Call for Action. J Pain Res. 2024;17:1967–78. doi: 10.2147/JPR.S464009 38828088 PMC11144433

[27] Lanini I, Amass T, Calabrisotto CS, Fabbri S, Falsini S, Adembri C, et al. The influence of psychological interventions on surgical outcomes: a systematic review. J Anesth Analg Crit Care. 2022;2(1):31. doi: 10.1186/s44158-022-00057-4 37386591 PMC10245433

[28] Bilginer C, Punduk M, Cetin A, Guleroglu FY, Erol N, Cim N. Factors influencing surgical anxiety and postoperative pain: a comprehensive evaluation of psychological and gynecological determinants. BMC Womens Health. 2025;25(1):103. doi: 10.1186/s12905-025-03623-4 40050846 PMC11887388

[29] Li M, Yan E, Saripella A, Alhamdah Y, Nabipoor M, Alibhai SMH, et al. Understanding preoperative concerns and attitudes towards prehabilitation in older surgical populations: A survey study. J Clin Anesth. 2025;105:111895. doi: 10.1016/j.jclinane.2025.111895 40479950

[30] Maniaci A, Lentini M, Vaira L, Lavalle S, Ronsivalle S, Rubulotta FM, et al. The Global Burden of Maxillofacial Trauma in Critical Care: A Narrative Review of Epidemiology, Prevention, Economics, and Outcomes. Medicina (Kaunas). 2025;61(5):915. doi: 10.3390/medicina61050915 40428873 PMC12113130

[31] Jaxa-Kwiatkowski A, Jaxa-Kwiatkowski M, Jaxa-Kwiatkowska K, Gerber H, Kubiak M, Łysenko L. Perioperative Nutritional and Metabolic Factors Affecting Surgical Outcomes in Head and Neck Cancer Free Flap Reconstruction: A Comprehensive Review. J Clin Med. 2025;14(11):3679. doi: 10.3390/jcm14113679 40507441 PMC12155434

[32] Coyle MJ, Main B, Hughes C, Craven R, Alexander R, Porter G, et al. Enhanced recovery after surgery (ERAS) for head and neck oncology patients. Clin Otolaryngol. 2016;41(2):118–26. doi: 10.1111/coa.12482 26083896

[33] Pramesh CS, Badwe RA, Bhoo-Pathy N, Booth CM, Chinnaswamy G, Dare AJ, et al. Priorities for cancer research in low- and middle-income countries: a global perspective. Nat Med. 2022;28(4):649–57. doi: 10.1038/s41591-022-01738-x 35440716 PMC9108683

[34] Whitcomb DC. Barriers and Research Priorities for Implementing Precision Medicine. Pancreas. 2019;48(10):1246–9. doi: 10.1097/MPA.0000000000001415 31688586 PMC6857715

[35] Smith DM, Figg WD. Evidence Regarding Pharmacogenetics in Pain Management and Cancer. Oncologist. 2023;28(3):189–92. doi: 10.1093/oncolo/oyac277 36718020 PMC10020807

[36] Ferreira do Couto ML, Fonseca S, Pozza DH. Pharmacogenetic Approaches in Personalized Medicine for Postoperative Pain Management. Biomedicines. 2024;12(4):729. doi: 10.3390/biomedicines12040729 38672085 PMC11048650

[37] Ginnane JF, Aziz S, Sultana S, Allen CL, McDougall A, Eddy KE, et al. The cost-effectiveness of preventing, diagnosing, and treating postpartum haemorrhage: A systematic review of economic evaluations. PLoS Med. 2024;21(9):e1004461. doi: 10.1371/journal.pmed.1004461 39269991 PMC11433145

[38] Moyo N, Madzimbamuto F. Teaching of chronic pain management in a low- and middle-income setting: a needs assessment survey. Pain Rep. 2019;4(1):e708. doi: 10.1097/PR9.0000000000000708 30801046 PMC6370143

