# Supplementary material for: Multimodal analgesia in resource-limited settings: A comparative analysis of postoperative pain management strategies in Pakistan
Source: PLOS Glob Public Health. 2025 Dec 19;5(12):e0005345. doi: 10.1371/journal.pgph.0005345 (PMC12716754; doi:10.1371/journal.pgph.0005345)
Supplement: S1 Table — (DOCX) [file pgph.0005345.s001.docx]

# S1 Table

Agent-level analgesic exposures by mutually exclusive group, with doses, routes, timing, and rescue use.

| Exposure Group | Agent (Generic Name) | Class | Route | Timing Window | Dosing | Dose Metric (Median [IQR]) | % Receiving Rescue | Notes |
| --- | --- | --- | --- | --- | --- | --- | --- | --- |
| Opioid-only | Morphine | Opioid | IV | 0–6 h | Bolus | 5 mg [4–6] | 12% | Rescue given within 24 h |
| Non-opioid-only | Paracetamol | Non-opioid | Oral | 0–6 h | Regular | 1 g [1–1] | 8% | Standard regimen |
| Regional + non-opioid | Bupivacaine | Regional | Peripheral block | Intraop | Single shot | 20 mL [20–20] | 5% | With diclofenac adjunct |
| True multimodal | Fentanyl + Paracetamol | Opioid + Non-opioid | IV + Oral | 0–6 h overlap | Infusion + Regular | Fentanyl 100 mcg/h; Paracetamol 1 g | 15% | Overlap documented |
