# Supplementary material for: Multimodal analgesia in resource-limited settings: A comparative analysis of postoperative pain management strategies in Pakistan
Source: PLOS Glob Public Health. 2025 Dec 19;5(12):e0005345. doi: 10.1371/journal.pgph.0005345 (PMC12716754; doi:10.1371/journal.pgph.0005345)
Supplement: S2 Table — (DOCX) [file pgph.0005345.s002.docx]

**S2 Table. Agent-level exposure details by analgesic strategy (N = 431)**

| **Exposure Group** | **Agents Used** | **Typical Dose (Median [IQR])** | **Route(s)** | **Timing Window** | **Dosing Schedule** | **% of Group (n)** |
| --- | --- | --- | --- | --- | --- | --- |
| **Opioid-only** | Morphine | 5 mg [4–10] | IV/IM | PACU–24 h | Bolus / PRN | 42.9% (51) |
|  | Tramadol | 100 mg [50–100] | IV/PO | 0–6 h, 6–24 h | Bolus / 8–12 hourly | 33.6% (40) |
|  | Fentanyl | 50–100 μg | IV | Intra-op / PACU | Bolus | 23.5% (28) |
| **Non-opioid-only** | Paracetamol | 1 g [1–2 g] | IV/PO | Intra-op; 0–6 h; 6–24 h | 6–8 hourly | 61.7% (71) |
|  | Diclofenac | 75 mg [50–100] | IM/IV | 0–6 h, 6–24 h | 12 hourly | 25.2% (29) |
|  | Gabapentin | 300 mg [300–600] | PO | Pre-op; 0–6 h | Once daily | 13.1% (15) |
| **Regional + Non-opioid adjuncts** | Bupivacaine | 0.25–0.5% (10–20 mL) | Regional (nerve block/infiltration) | Intra-op | Single-shot | 41.2% (28) |
|  | Lidocaine | 1–2% (5–10 mL) | Local infiltration | Intra-op | Single-shot | 17.6% (12) |
|  | Paracetamol | 1 g | IV/PO | 0–6 h, 6–24 h | 6–8 hourly | 41.2% (28) |
| **True multimodal** | Morphine + Paracetamol | 5 mg + 1 g | IV/PO | 0–6 h overlap | Bolus + 6 hourly | 36.4% (47) |
|  | Tramadol + Diclofenac | 100 mg + 75 mg | IV/IM | 0–6 h; 6–24 h | 8–12 hourly | 31.8% (41) |
|  | Fentanyl + NSAID (Ketorolac 30 mg) | 50 μg + 30 mg | IV | Intra-op / PACU overlap | Bolus | 18.6% (24) |
|  | Morphine + Regional block (Bupivacaine 0.25–0.5%) | 5 mg IV + 10 mL block | PACU + intra-op overlap | Bolus + single-shot | 13.2% (17) |  |

**Supplementary Notes**

- *Rescue therapy*: 28.5% of patients (n = 123) required rescue doses, most commonly IV morphine (5 mg bolus) or tramadol (50 mg). Rescue therapy was recorded as a separate variable; if administered within 24 h and overlapping with another class, patients were reclassified into **true multimodal**.
- *Timing windows*: defined as intraoperative, 0–6 h, 6–24 h, >24 h.
- *Dosing schedules*: bolus, continuous infusion, PRN, or fixed-interval (6–12 hourly).
- *Side-effects*: reported within 72 h but not attributed to individual drugs due to multimodal overlap.
