# Supplementary material for: Multimodal analgesia in resource-limited settings: A comparative analysis of postoperative pain management strategies in Pakistan
Source: PLOS Glob Public Health. 2025 Dec 19;5(12):e0005345. doi: 10.1371/journal.pgph.0005345 (PMC12716754; doi:10.1371/journal.pgph.0005345)
Supplement: S3 Table — (DOCX) [file pgph.0005345.s003.docx]

**S3 Table**

Paste as **Supplementary Table S3** and edit/extend rows to match the opioids used at your site.

**Supplementary Table S3. Route-appropriate equianalgesic conversion factors to morphine milligram equivalents (MME), and computation examples**

| **Opioid (route)** | **Equianalgesic factor to MME*** | **Example dose** | **MME calculation example** |
| --- | --- | --- | --- |
| **Morphine IV/IM** | 1.0 per mg | 5 mg IV bolus | 5 mg × 1.0 = **5 MME** |
| **Morphine PO** | 0.5 per mg (PO ≈ half of IV potency) | 10 mg PO | 10 × 0.5 = **5 MME** |
| **Fentanyl IV** | 0.1 mg (100 µg) ≈ 10 mg IV morphine ⇒ **0.1 mg fentanyl = 10 MME** → factor **100 µg = 10 MME** | 150 µg IV | (150 µg / 100 µg) × 10 = **15 MME** |
| **Tramadol PO/IV** | 0.1 per mg | 100 mg IV/PO | 100 × 0.1 = **10 MME** |
| **Hydromorphone IV** | 1.5 mg ≈ 10 mg IV morphine ⇒ factor **6.67 per mg** | 0.5 mg IV | 0.5 × 6.67 = **3.3 MME** |
| **Oxycodone PO** | 1.5 per mg (PO) | 5 mg PO | 5 × 1.5 = **7.5 MME** |
| **Pethidine/Meperidine IV** (if used) | 75 mg ≈ 10 mg IV morphine ⇒ **0.133 per mg** | 25 mg IV | 25 × 0.133 = **3.3 MME** |
| **Neuraxial morphine (IT/epidural)** | Reported as morphine mg; include in primary analysis and **exclude in sensitivity** | 0.2 mg IT | 0.2 × 1.0 = **0.2 MME** |

*Factors reflect commonly used perioperative equianalgesic approximations; variability exists across sources and patient contexts. All conversions were applied consistently across patients/time windows; sensitivity analyses excluding neuraxial opioid are reported.

**Computation templates**

1. **PCA with basal + demand (IV morphine)**

- Basal = 0.5 mg/h × 12 h = **6 mg** (6 MME)
- Demand = 1 mg per delivery × 8 successful deliveries = **8 mg** (8 MME)
- **Total PCA MME (0–24 h)** = 6 + 8 = **14 MME**

1. **Continuous infusion (IV fentanyl)**

- 25 µg/h × 10 h = **250 µg** total
- MME = (250 µg / 100 µg) × 10 = **25 MME**

1. **Mixed intra-op + ward doses**

- Intra-op fentanyl 100 µg = **10 MME**
- PACU morphine 4 mg IV = **4 MME**
- Ward tramadol 100 mg IV = **10 MME**
- **Total 0–24 h MME** = 10 + 4 + 10 = **24 MME**
