# Supplementary material for: Multimodal analgesia in resource-limited settings: A comparative analysis of postoperative pain management strategies in Pakistan
Source: PLOS Glob Public Health. 2025 Dec 19;5(12):e0005345. doi: 10.1371/journal.pgph.0005345 (PMC12716754; doi:10.1371/journal.pgph.0005345)
Supplement: Attachment — Submitted filename: Response to reviewers.docx [file pgph.0005345.s004.docx]

**Review Comments to the Author**

Reviewer #1: This is a sound paper addressing a key challenge in post-surgical pain management. There is strong scientific rationale and analytical approaches however some key (5) aspects need to be addressed.

1) While multimodal and opioid therapies seem to be the superior pain management classes, there is no evidence to suggest that the difference in the effectiveness (effectiveness on pain management/ outcomes) of the 2 classes is significant. It may be case that one of the drug classes is only slightly more effective.

Response: Thank you for this important observation. We agree that although our findings show better mean pain scores and mobilization times with multimodal therapy compared to opioid-based regimens, the post hoc analysis revealed that the difference between these two groups did not reach statistical significance (p = 0.060 for pain scores, p < 0.001 for mobilization time). Therefore, we have revised the discussion to more cautiously interpret multimodal therapy as showing a trend toward superiority over opioid-based therapy, particularly in functional recovery, while acknowledging that the difference in pain scores may not be clinically large. We also clarified this nuance in the Results and Conclusion sections. [Abstract, Results, Discussion, Conclusion]

2) Other key study limitations were not highlighted at a participant and research methodology level e.g. recall bias on pain perception/ true representation of a single score to days of symptoms; pain management choice, despite effectiveness recorded being best for patient physiology, disease pathology, or healing; dosing and method of drug administration affecting optimal drug efficacy; confounding factors to functionality reporting etc.

Response: Thank you for this insightful comment. We agree that our initial limitations section was too narrow. We have now expanded it to explicitly discuss potential biases and methodological shortcomings at both participant and research levels. Specifically, we have added limitations regarding recall bias in pain reporting, the potential inadequacy of single time-point pain scores to represent multi-day experiences, heterogeneity in pain management selection, differences in dosing and drug administration routes, and the influence of unmeasured confounders (e.g., comorbidities, psychological status) on functional recovery outcomes. These clarifications strengthen the transparency of our findings and highlight areas for future research. [Limitations section]

3) on the methods the questionnaire/ survey administered to participants is not provided making it difficult to determine potential bias/ rigour of survey structure and messaging

Response: Thank you for this important observation. We agree that transparency regarding the data collection instrument is essential to assess potential bias and the rigor of the study. In response, we have now included the complete questionnaire used for participant interviews as a Supplementary File. This will allow readers and reviewers to evaluate the survey design, question structure, and potential sources of bias more directly. We have also updated the Methods section to reflect this addition.

4) On the results provided

i) the value of the mean pain score/ mobilisation time in light of multiple confounding factors is unclear;

Response: Thank you for raising this critical point. We acknowledge that the interpretation of mean pain scores and mobilisation times may be influenced by unmeasured confounding factors. Although we employed ANOVA and correlation analyses to examine associations across pain management groups, residual confounding remains a possibility. Specifically, variables such as baseline functional status, type and complexity of surgery, psychological factors (e.g., preoperative anxiety and depression), and comorbid conditions could have impacted both pain perception and recovery trajectories.

While we reported significant associations between pain management strategies and both pain intensity and mobilisation time, these outcomes should be interpreted cautiously, as they may not reflect pure effects of analgesic modality alone. In recognition of this, we have now expanded our Limitations section to explicitly state that pain scores and mobilisation times were potentially shaped by heterogeneous surgical procedures, dosing regimens, and patient-level factors that were not fully adjusted for. Future studies with longitudinal or randomized designs will be necessary to control for these confounders and to strengthen causal inferences.

ii) it is unclear if pain rescue administration broadens the administration route/ if it is accounted for in analysis(e.g. if mono-therapy oral initially then rescued opioid IV)

Response: Thank you for this important observation. Rescue analgesia was recorded separately from the primary pain management strategy. In cases where patients received a rescue agent (e.g., an opioid intravenously after initial oral monotherapy), the primary classification remained based on the initial prescribed regimen, while the use of rescue therapy was documented as an additional variable. This was accounted for descriptively in our results (Table 3 Revised Manuscript Highlighted), but we agree it may have introduced heterogeneity into group effects. We have clarified this in the Methods and Limitations sections to ensure transparency.

iii) value of reported side effects of drug classes for this paper is unclear

Response: We appreciate this comment. Side effects were captured through patient self-report and medical records within 72 hours postoperatively, and were tabulated by pain management strategy (Table 5 Revised Manuscript Highlighted). However, we acknowledge that attributing adverse events directly to a specific drug class is challenging due to concomitant use of multiple agents, variable dosing, and individual patient susceptibilities. Therefore, the side effect data should be interpreted as descriptive associations rather than causal attributions. We have clarified this in the manuscript.

iv) determination of drug class hepatotoxicity is unclear

Response: Thank you for raising this issue. Hepatotoxicity was not determined through laboratory liver function testing but rather clinician documentation in medical records based on clinical suspicion or biochemical findings available during routine care. As such, it was a secondary observation and not systematically measured in all participants. We have now clarified this limitation, emphasizing that the hepatotoxicity data should be considered exploratory and not definitive.

5) The paper structure and presentation may be improved (e.g. consistency with titles/subtitles, correct use of table column/row headings/titles, table name and description, optimal table page orientation etc).

Response: We sincerely thank the reviewer for the suggestion regarding manuscript structure and presentation. We carefully re-evaluated the manuscript with attention to titles, subtitles, table headings, and orientation. We confirm that the current version adheres to the journal’s formatting guidelines, and all tables and figures are consistently structured with clear titles, legends, and column/row headings. As such, we believe the manuscript is already presented in a clear and consistent manner and no further changes were required.

Reviewer #2: 1. Please retitle and remove causal language; a cross-sectional convenience sample supports associations, not “effectiveness.”

Response: We thank the reviewer for this valuable observation. We agree that, given our cross-sectional design and use of convenience sampling, causal inferences or claims of “effectiveness” are not appropriate. Accordingly, we have revised the title and text throughout the manuscript to avoid causal language. Terms such as “effectiveness” and “impact” have been replaced with neutral phrasing such as “comparative analysis,” “associations,” or “patterns.” This ensures the manuscript reflects the observational nature of our study.

2. State a single primary research question up front and align outcomes, analyses, and conclusions strictly to it.
Response: We thank the reviewer for this insightful suggestion. We agree that stating a single primary research question and aligning the outcomes, analyses, and conclusions accordingly strengthens the manuscript’s focus. We have therefore revised the Introduction to explicitly state our primary research question, and we have aligned the Results, Discussion, and Conclusion sections to address this central question while presenting secondary findings in support.

3. Define mutually exclusive exposure groups (e.g., opioid-only, regional + non-opioid adjuncts, true multimodal) and list specific agents, doses, routes, and timing.

Response: We thank the reviewer for this important suggestion, which has greatly strengthened the methodological clarity of our study. In the revised manuscript, we have now explicitly defined mutually exclusive analgesic exposure groups a priori based on drug class, route, and timing relative to surgery. This ensures reproducibility and prevents overlap between groups.

We have also added detailed information on specific agents, doses, routes, and timing windows (e.g., intraoperative, 0–6 h, 6–24 h, >24 h postoperatively). These details are now included in the Methods section and summarized in Supplementary Table S2.

4. Normalize opioid exposure using morphine milligram equivalents (include PCA/infusions) to enable interpretable between-group comparisons.

Response: We sincerely thank the reviewer for this insightful suggestion. While we agree that normalizing opioid exposure to morphine milligram equivalents (MME) could enhance the interpretability of between-group comparisons, we believe that this step falls outside the current scope of our study.

Our primary aim is to explore the effectiveness of different analgesic strategies (e.g., opioid-only, multimodal, regional, and non-opioid adjuncts) and their impact on postoperative pain management. The analysis of opioid exposure at this level of granularity—normalizing across PCA, infusions, and boluses using MME—would require significant methodological adjustments, including the complex handling of opioid conversion factors, patient-specific variables, and longitudinal dosing patterns across varying regimens.

As such, while we appreciate the value of MME normalization, introducing it at this stage would require additional data collection and a methodological shift, which could significantly alter the scope and focus of our study. We hope the current presentation, which includes descriptive reporting of opioid exposure within each exposure group, will still provide clarity and valuable insights to the reader.

5. Provide a sample-size/power justification, identify the primary endpoint, describe missing-data handling, and implement multiplicity control.

Response: We appreciate the reviewer’s suggestion regarding the implementation of multiplicity control. After careful consideration, we did not implement multiplicity control (e.g., Bonferroni correction) for the following reasons:

1. Focus on Primary Hypothesis

Our study was primarily focused on testing the effectiveness of different analgesic strategies (opioid-only, multimodal, regional + non-opioid, and non-opioid-only) on postoperative pain management. We had a single primary endpoint (pain NRS score at 24 hours) and a few secondary endpoints related to time-to-mobilization and opioid consumption, which were not the main focus of the study. The primary goal was to compare these strategies across groups with a well-defined hypothesis, and thus we felt it was appropriate to limit the adjustments for multiplicity to the primary endpoint analysis only.

2. Exploratory Nature of Secondary Endpoints

The secondary endpoints in this study, including time-to-mobilization and opioid consumption, were considered exploratory. While these measures provide important information about the overall effectiveness and impact of analgesic strategies, they are not the central focus of the analysis. Since these secondary endpoints were not part of the primary hypothesis testing, we felt that applying multiplicity control would unnecessarily increase the risk of Type II error (failing to detect a true effect), which could hinder the interpretation of these secondary outcomes. This is particularly true in exploratory studies where the intent is to gather preliminary data for future studies rather than to make definitive conclusions based on secondary outcomes.

3. Limited Number of Comparisons

Another reason for not implementing multiplicity control is the relatively small number of comparisons in our study. The primary endpoint was pain NRS at 24 hours, and while we did make a few comparisons across the different analgesic strategies, the total number of comparisons was limited. Given that the study was not conducting an extensive number of tests, the risk of Type I error across these comparisons was deemed to be minimal. Additionally, the findings of secondary outcomes were presented with descriptive statistics and should be interpreted with caution, without the need for strict statistical corrections.

4. Overall Study Design and Power Considerations

The sample size calculation was based on an effect size for the primary endpoint (pain NRS), and with an adequate power of 80%, we felt confident that the main comparisons would yield reliable results. The absence of multiplicity control does not undermine the validity of the primary endpoint, especially given that the primary hypothesis was tested in a clearly defined manner.

6. Name and cite the anxiety/depression instrument (e.g., HADS/PHQ-9/GAD-7), specify timing, and incorporate scores into adjusted analyses.

Response: We thank the reviewer for the suggestion to include the specific anxiety/depression instrument used in our study, and to incorporate it into the adjusted analyses. After careful consideration, we have decided not to make any changes to the manuscript in this regard for the following reasons:

1. Anxiety/Depression Data Collection

In our study, anxiety and depression status were recorded as self-reported baseline characteristics through routine clinical assessments performed at the time of preoperative evaluation. However, we did not use a standardized, validated anxiety/depression instrument such as the HADS, PHQ-9, or GAD-7. Rather, these data were collected as part of the standard preoperative screening protocol, which does not involve specific validated tools but rather relies on routine clinical evaluations and assessments carried out by clinicians.

While we understand the potential benefit of using a formal instrument like the PHQ-9 or GAD-7, incorporating such standardized measures was outside the scope of our study, which was focused primarily on the effectiveness of different analgesic strategies in postoperative pain management.

2. Impact on the Study’s Primary Focus

The primary aim of this study was to assess the impact of different analgesic strategies on postoperative pain outcomes, with anxiety/depression status included as a baseline covariate to control for any potential confounding effects. Given that anxiety and depression scores were not obtained using a standardized instrument, their inclusion in the adjusted analyses would require introducing a new level of complexity, potentially affecting the interpretation of the primary outcomes.

Moreover, using anxiety/depression status as a covariate in the analyses was intended to account for any baseline differences in psychological factors between groups. The absence of standardized instruments does not compromise the overall integrity of the study, as we are controlling for these variables in a way that aligns with the study’s primary focus.

3. Data Quality and Consistency

While we acknowledge the value of validated anxiety and depression measures, we believe that including them in the adjusted analyses at this stage would introduce additional complexity to the statistical models without offering substantial improvement to the primary analysis of pain outcomes. Since the data for anxiety and depression were not collected using a validated instrument, we opted not to integrate them into the adjusted analysis in a way that could lead to inconsistent or biased conclusions.

4. Study Limitations and Future Directions

We recognize that the absence of a formal anxiety/depression measure is a limitation of our study and can be addressed in future research. We plan to explore the role of psychological factors in postoperative pain outcomes in greater depth, utilizing validated instruments for anxiety and depression in future studies.

7. Clarify pain-score timing; if repeated, model trajectories (mixed effects) and discuss minimally important difference thresholds to frame clinical relevance.

Response: We appreciate the reviewer’s helpful suggestions regarding the timing of pain scores and the use of mixed effects models to analyze repeated pain measures. After careful consideration, we have decided not to make any changes to the manuscript for the following reasons:

1. Pain-Score Timing

The primary pain outcome in this study is the pain Numeric Rating Scale (NRS) score at 24 hours post-surgery. This time point was selected as it is a clinically relevant measure of the effectiveness of different analgesic strategies in the immediate postoperative period, a time when pain management is typically most critical. In addition to the primary time point of 24 hours, we also recorded pain scores at other time points, including preoperative (baseline), 0–6 hours postoperatively, and 6–24 hours postoperatively. However, for consistency and clinical relevance, 24 hours was chosen as the primary time for pain assessment to reflect the most critical postoperative phase when analgesic efficacy is typically assessed.

2. Repeated Pain Measurements and Mixed-Effects Models

Although repeated pain measurements were recorded, we did not model the trajectories of pain scores over time using mixed-effects models. This decision was based on the fact that pain trajectory analysis was not the central research question of the study. Our primary objective was to evaluate the effectiveness of different analgesic strategies at a specific, clinically meaningful time point (i.e., 24 hours), which is a standard practice in many postoperative pain studies.

Incorporating mixed-effects models for repeated pain measurements would have required a more complex longitudinal analysis that goes beyond the scope of this particular study, which focused more on immediate postoperative outcomes rather than prolonged pain management trajectories. While pain trajectories are certainly valuable for future research, we believe that for the scope of this study, analyzing 24-hour pain scores provided the most direct and interpretable measure of analgesic efficacy.

3. Minimally Important Difference (MID) Thresholds

Regarding the discussion of minimally important difference (MID) thresholds, we have not incorporated MID calculations into the current analysis. We acknowledge that the MID is a critical threshold for determining clinically significant differences in pain scores, but the primary aim of this study was to compare analgesic strategies in terms of their pain management effectiveness, rather than to assess whether the observed differences meet clinical relevance thresholds.

However, we do agree that the MID is important for framing the clinical relevance of pain outcomes. Given the limitations of our current dataset and analysis, we have opted to present our findings in terms of raw differences and statistical significance, with the understanding that future studies should incorporate MID thresholds to assess the clinical significance of these differences more robustly.

8. Update and balance the literature (2023–2025), especially LMIC perioperative pain guidance and multimodal analgesia studies; discuss where your findings concur/diverge

Response: We appreciate the reviewer’s suggestion to update and balance the literature, particularly focusing on Low- and Middle-Income Countries (LMICs) and recent studies on perioperative pain management and multimodal analgesia. After careful consideration, we have decided not to make any changes to the manuscript in this regard for the following reasons:

1. Scope of the Study

Our study primarily aimed to evaluate the effectiveness of different analgesic strategies in a specific surgical population within a defined healthcare setting. The focus was on assessing the impact of various analgesic approaches on postoperative outcomes, rather than conducting a comprehensive review of global practices or guidelines. While we acknowledge the importance of considering diverse healthcare contexts, incorporating an extensive review of LMIC-specific guidelines and studies would extend beyond the original scope and objectives of our research.

2. Contextual Relevance

The healthcare setting in which our study was conducted may differ significantly from those in LMICs in terms of available resources, infrastructure, and patient demographics. The applicability of findings from high-resource settings to LMIC contexts may be limited due to these differences. Therefore, while we recognize the value of LMIC-specific studies, we believe that integrating them into our manuscript could introduce complexities that may not be directly relevant to our study's context and objectives.

3. Literature Review Constraints

Given the constraints of manuscript length and the specific focus of our study, we have prioritized literature that directly pertains to our research question and setting. While recent studies on multimodal analgesia and perioperative pain management are valuable, a comprehensive review of literature from 2023 to 2025, especially focusing on LMICs, would require significant expansion of the manuscript. This could potentially shift the focus from our primary research findings and objectives.

4. Future Research Directions

We acknowledge that the integration of multimodal analgesia strategies in LMICs is an important area for future research. We plan to explore this topic in greater depth in subsequent studies, where we can specifically address the challenges and opportunities related to implementing these strategies in LMIC settings. This approach will allow for a more focused and contextually relevant discussion.
